# A Continuum Mathematical Model of Substrate-Mediated Tissue Growth

**DOI:** 10.1007/s11538-022-01005-7

**Published:** 2022-03-02

**Authors:** Maud El-Hachem, Scott W. McCue, Matthew J. Simpson

**Affiliations:** School of Mathematical Sciences, Queensland University of Technology, Brisbane, Australia

**Keywords:** Tissue engineering, Travelling wave, Fisher-KPP, Porous-Fisher, Diffusion, Logistic growth

## Abstract

We consider a continuum mathematical model of biological tissue formation inspired by recent experiments describing thin tissue growth in 3D-printed bioscaffolds. The continuum model, which we call the *substrate model*, involves a partial differential equation describing the density of tissue, ${\hat{u}}(\hat{{\mathbf {x}}},{\hat{t}})$ that is coupled to the concentration of an immobile extracellular substrate, ${\hat{s}}(\hat{{\mathbf {x}}},{\hat{t}})$. Cell migration is modelled with a nonlinear diffusion term, where the diffusive flux is proportional to ${\hat{s}}$, while a logistic growth term models cell proliferation. The extracellular substrate ${\hat{s}}$ is produced by cells and undergoes linear decay. Preliminary numerical simulations show that this mathematical model is able to recapitulate key features of recent tissue growth experiments, including the formation of sharp fronts. To provide a deeper understanding of the model we analyse travelling wave solutions of the substrate model, showing that the model supports both sharp-fronted travelling wave solutions that move with a minimum wave speed, $c = c_{\mathrm{min}}$, as well as smooth-fronted travelling wave solutions that move with a faster travelling wave speed, $c > c_{\mathrm{min}}$. We provide a geometric interpretation that explains the difference between smooth and sharp-fronted travelling wave solutions that is based on a slow manifold reduction of the desingularised three-dimensional phase space. In addition, we also develop and test a series of useful approximations that describe the shape of the travelling wave solutions in various limits. These approximations apply to both the sharp-fronted and smooth-fronted travelling wave solutions. Software to implement all calculations is available at GitHub.

**Supplementary Information:**

The online version contains supplementary material available at 10.1007/s11538-022-01005-7.

## Introduction

**Fig. 1:**
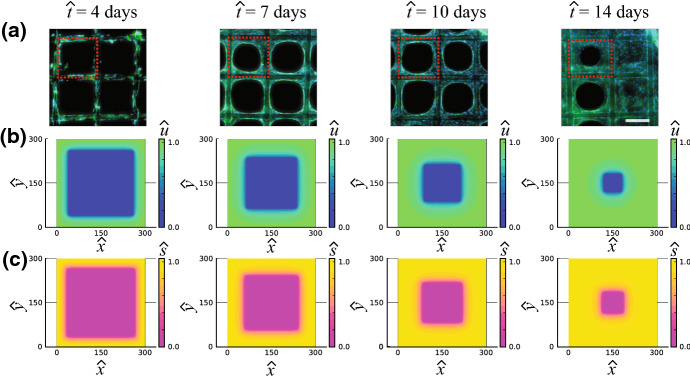
Experimental and simulated osteoblast tissue formation within a square-shaped 3D-printed pore. (**a**) Composite fluorescence microscopy images of pore bridging experiments (Buenzli et al. 2020; Browning et al. 2021). Cell nuclei are shown in blue; tissue and cytoskeleton are shown in green. Each subfigure shows four adjacent square pores, each with side length of ${\hat{L}}=300$ $\upmu$m. Images are shown at various times, ${\hat{t}} = 4, 7, 10$ and 14 days, as indicated. For clarity, in each subfigure we outline the border of the upper-left pore (red dashed). Experimental images are reproduced from Buenzli et al. (2020) with permission. (**b**)–(**c**) Numerical solution of Eqs. (1)–(2) on a square domain with side length ${\hat{L}}=300$ $\upmu$m. (**b**) Evolution of ${\hat{u}}$. (**c**) Evolution of ${\hat{s}}$. Each column of the figure corresponds to ${\hat{t}} = 4, 7, 10$ and 14 days, as indicated. Parameter values for the mathematical model are ${\hat{D}}=300$ $\upmu$m$^2$/day, ${\hat{\lambda }} = 0.6$ /day, ${\hat{K}}_u = 1$ cells/$\upmu$m$^2$, ${\hat{K}}_s = 1$ mol/$\upmu$m$^2$, ${\hat{r}}_1 = 1$ mol/(cells day), ${\hat{r}}_2 = 1$ /day. The numerical solution of (1)–(2) is obtained on a $101\times 101$ mesh. Temporal integration is performed with uniform time steps of duration $\Delta {\hat{t}} = 1\times 10^{-2}$ day (colour figure online)

Over the last decade, tissue engineering has been revolutionised through the use of 3D printing technologies that produce 3D bioscaffolds upon which $\textit{in vitro}$ tissues can be grown in biologically realistic geometries (Ambrosi et al. 2019; Dzobo et al. 2018). *In vitro* tissues grown on 3D scaffolds are more reproducible and more biologically realistic than tissues grown in traditional two-dimensional tissue culture (Lanaro et al. 2021). The experimental images in Fig. 1a show the evolution of thin 3D tissues that are produced by seeding a 3D-printed scaffold with osteoblast precursor cells (Buenzli et al. 2020; Browning et al. 2021). In this experiment, cells are seeded onto the perimeter of 3D-printed square shaped pores, where each pore has sides of approximately 300 $\mu$m in length. Each subfigure in Fig. 1a shows four adjacent pores. As the experiment proceeds, individual cells migrate off the scaffold into the pore, and then combined cell migration and cell proliferation lead to the formation of a sharp-fronted tissue profile that invades into the pore. This process eventually forms a thin tissue that closes or *bridges* the pore after approximately 14 days (Buenzli et al. 2020; Browning et al. 2021). A notable feature of these experiments is that tissue formation involves a well-defined moving front that is very obvious in Fig. 1a. Closer inspection of these experimental images shows that cells not only migrate and proliferate during the pore bridging process, but they also produce an extracellular medium that is laid down onto the surface of the pore (Lanaro et al. 2021).

Continuum mathematical models of tissue formation have a long history, with many early examples based on the classical Fisher-KPP model (Ablowitz and Zeppetella 1979; Canosa 1973; Fisher 1937; Kolmogorov et al. 1937). The Fisher-KPP model describes cell migration using a one-dimensional linear diffusion term, and cell proliferation is treated using a logistic source term. Different types of tissue formation experiments have been successfully modelled using the Fisher-KPP model (Maini et al. 2004; Jin et al. 2016, 2017; Warne et al. 2019) or two-dimensional extensions (Sherratt and Murray 1990; Swanson et al. 2003). While these studies show that simple mathematical models based on the Fisher-KPP framework successfully capture certain features of tissue formation, there are several well-known limitations that can be addressed by considering extensions of that model (Murray 2002). One such criticism is that the linear diffusion term in the Fisher-KPP model leads to smooth density profiles that do not represent well-defined fronts, such as those we see in Fig. 1a.

One way to overcome this limitation is to work with the Porous-Fisher model where the linear diffusion term is generalised to a degenerate nonlinear diffusion term with a power law diffusivity (Fadai and Simpson 2020; Sanchez and Maini 1994; Sengers et al. 2007; Witeslki 1994; Witelski 1995). While the Porous-Fisher model leads to sharp-fronted density profiles, this approach introduces a separate complication of having to justify the choice of the exponent in the power law diffusivity (Jin et al. 2016; McCue et al. 2019; Sherratt and Murray 1990; Simpson et al. 2011; Warne et al. 2019). A further weakness of both the Fisher-KPP and Porous-Fisher models is that they deal with a single species, such as a density of cells, and do not explicitly describe how the population of cells invades into surrounding cells, or interacts with the surrounding environment. This second limitation has been addressed by introducing more complicated mathematical models, such as the celebrated Gatenby–Gawlinski model of tumour invasion (Gatenby and Gawlinski 1996), which explicitly describes how a population of tumour cells degrades and invades into a population of surrounding healthy tissue by explicitly modelling both populations and their interactions. Since the Gatenby–Gawlinski framework was proposed in 1996, subsequent studies have since analysed the relationship between individual-level mechanisms and the resulting population-level continuum descriptions (Painter et al. 2003), calibrating these mathematical models to match experimental measurements of melanoma invasion (Browning et al. 2019), as well as analysing travelling wave solutions of these types of multi-species mathematical models (Colson et al. 2021; El-Hachem et al. 2021b; Gallay and Mascia 2021).

In this work, we study a continuum mathematical model of cell invasion that is motivated by the experimental images in Fig. 1a. The mathematical model explicitly describes the evolution of the cell density, ${\hat{u}}(\hat{{\mathbf {x}}},{\hat{t}})$, and the density of substrate produced by the cells, ${\hat{s}}(\hat{{\mathbf {x}}},{\hat{t}})$, giving rise to a coupled system of nonlinear partial differential equations (PDEs). We first explore numerical solutions of the mathematical model in two spatial dimensions to mimic the same patterns of tissue development that we see in the experimental images in Fig. 1a.

Within this modelling framework, it is natural for us to ask how the duration of time required for the pore to close is affected by the dynamics of substrate deposition and decay. We address this question by nondimensionalising the mathematical model and numerically exploring travelling wave solutions in one dimension. Not only does travelling wave analysis of the mathematical model has a direct link to the application in question, we note that travelling wave analysis provides mathematical insight into various models of invasion with applications including tissue engineering (Landman and Cai 2007), directed migration (Krause and Van Gorder 2020), disease progression (Strobl et al. 2020), and various applications in ecology (Hogan and Myerscough 2017; El-Hachem et al. 2021a). Our preliminary numerical explorations suggest that, similar to the well-known Porous-Fisher model, the substrate model supports both sharp-fronted and smooth travelling wave solutions. Working in three-dimensional phase space, we show that travelling wave solutions exist for all wave speeds $c \ge c_{\mathrm{min}}$, where $c_{\mathrm{min}} > 0$ is some minimum wave speed, and we provide a geometric argument based on a slow manifold reduction to distinguish between sharp-fronted travelling wave solutions that move with the minimum speed $c_{\mathrm{min}}$, from smooth travelling wave solutions that move faster than the minimum speed, $c > c_{\mathrm{min}}$. The three-dimensional phase space arguments are supported by some analysis of the time-dependent PDE problem where we show how the long-time travelling wave speed relates to the initial decay rate of the cell density. All phase-space and time-dependent PDE analyses throughout this work are supported by detailed numerical simulations of the full time-dependent PDE model. For completeness we also present various perturbation solutions that give accurate mathematical expressions describing the shape of the travelling waves profiles in various limits.

Overall, we show that the substrate invasion model can be viewed as bridge between the relatively simple Porous-Fisher model and more detailed mathematical models of biological invasion. The substrate model supports various types of travelling wave solutions that are reminiscent of travelling wave solutions of the Porous-Fisher model, but the analysis of these travelling wave solutions is quite different, as we shall now explore.

## Results and Discussion

In this work all dimensional variables and parameters are denoted with a circumflex, and nondimensional quantities are denoted using regular symbols.

### Biological Motivation

Following Buenzli et al. (2020), we consider the following minimal model of cell invasion

1 $$\begin{aligned}&\dfrac{\partial {\hat{u}}}{\partial {\hat{t}}}={\hat{D}} \nabla \cdot {\left( \dfrac{{\hat{s}}}{{\hat{K}}_s} \nabla {\hat{u}}\right) } +{\hat{\lambda }} {\hat{u}}\left( 1-\dfrac{{\hat{u}}}{{\hat{K}}_u}\right) ,&\hat{{\mathbf {x}}} \in \Omega , \end{aligned}$$

2 $$\begin{aligned}&\dfrac{\partial {\hat{s}}}{\partial {\hat{t}}}= {\hat{r}}_1 {\hat{u}} - {\hat{r}}_2 {\hat{s}},&\hat{{\mathbf {x}}} \in \Omega , \end{aligned}$$

where ${\hat{u}}(\hat{{\mathbf {x}}},{\hat{t}}) \ge 0$ is the density of cells, ${\hat{s}}(\hat{{\mathbf {x}}},{\hat{t}}) \ge 0$ is the substrate concentration, ${\hat{D}}>0$ is the cell diffusivity and ${\hat{\lambda }}>0$ is the cell proliferation rate. This model assumes that cells produce an adhesive and immobile substrate at rate ${\hat{r}}_1>0$ and that the substrate decays at a rate ${\hat{r}}_2 > 0$. We assume that the carrying capacity density of cells is ${\hat{K}}_u>0$ and that a typical maximum substrate density is ${\hat{K}}_s>0$. The key feature of this mathematical model is that the diffusive flux of cells is proportional to the substrate density, ${\hat{s}}$. This assumption couples the cell density to the substrate concentration in a way that the diffusive flux vanishes when ${\hat{s}}=0$. In this model the evolution of the cell density is affected by the substrate through the cell migration term, without any direct coupling in the cell proliferation term. This assumption is consistent with recent two-dimensional studies that explored how different surface coatings affect combined cell migration and cell proliferation in wound healing assays (Jin et al. 2020). This work showed that different surface coatings have a dramatic impact on cell migration, whereas cell proliferation is less sensitive.

In this modelling framework we make use of the fact that the tissues produced in the experiments in Fig. 1a are thin; the horizontal length scale is approximately 300 $\mu$m whereas the depth of tissue is approximately one cell diameter only, which is around 10–20 $\mu$m. In this setting it is appropriate to use a depth-averaged modelling framework where variations in the vertical direction are implicit, rather than being explicitly described (Simpson 2009).

We begin by considering Eqs. (1)–(2) on a two-dimensional square-shaped domain, $\Omega = \{({\hat{x}},{\hat{y}}):0 \le {\hat{x}} \le {\hat{L}}, 0 \le {\hat{y}} \le {\hat{L}}\}$ to match the geometry of the experiments in Fig. 1a. For simplicity we work with Dirichlet boundary conditions by setting ${\hat{u}}={\hat{K}}_u$ along all boundaries, with spatially uniform initial conditions ${\hat{u}} = {\hat{s}} = 0$, at ${\hat{t}}=0$. A numerical solution of Eqs. (1)–(2) in Fig. 1b, c shows the evolution of ${\hat{u}}$ and ${\hat{s}}$, respectively. Full details of the numerical methods used to solve Eqs. (1)–(2) are given in Supplementary Material. While these initial conditions are inconsistent with the boundary data, our numerical results are grid-independent, suggesting that this initial discontinuity is regularised in the usual way. The evolution of ${\hat{u}}$ in Fig. 1b shows that the model predicts the sharp-fronted tissue growth that qualitatively matches the spatial and temporal patterns observed in the experiment. The evolution of ${\hat{s}}$ in Fig. 1c shows that the invading cell density profile is associated with an invading substrate profile. The coupling between the spatial and temporal distribution of the tissue and the underlying substrate is similar to that observed in the experiments (Lanaro et al. 2021). Given this experimental motivation we will now set about analysing the mathematical model to provide insight into how the substrate dynamics affect the speed of invasion.

### One-Dimensional Numerical Exploration

For the purpose of studying travelling wave solutions of the substrate model we rewrite Eqs. (1)–(2) in the one-dimensional Cartesian coordinate system. Introducing the following dimensionless quantities: $u={\hat{u}} /{\hat{K}}_u$, $s={\hat{s}} /{\hat{K}}_s$, $x = {\hat{x}}\sqrt{{\hat{\lambda }}/{\hat{D}}}$, $t = {\hat{\lambda }} {\hat{t}}$, $r_1 = {\hat{r}}_1{\hat{K}}_u/({\hat{\lambda }}{\hat{K}}_s)$ and $r_2 = {\hat{r}}_2/{\hat{\lambda }}$, gives the following non-dimensional model

3 $$\begin{aligned}&\dfrac{\partial u}{\partial t}= \dfrac{\partial }{\partial x}\left( s \dfrac{\partial u}{\partial x}\right) + u(1-u),&0< x < \infty \end{aligned}$$

4 $$\begin{aligned}&\dfrac{\partial s}{\partial t}= r_1 u - r_2 s,&0< x < \infty , \end{aligned}$$

5 $$\begin{aligned}&\dfrac{\partial u(0,t)}{\partial x}=0, \qquad \text {and} \quad \ u(x,t) \rightarrow 0, \ x \rightarrow \infty . \end{aligned}$$

This dimensionless model involves just two free parameters that relate to the rate of substrate production and the rate of substrate decay, $r_1$ and $r_2$, respectively. Note that Eq. (4) does not involve any spatial derivatives so there is no need to specify any boundary conditions for *s*.

In this study we will consider two different types of initial conditions: (i) a biologically realistic initial condition describing the situation where the initial cell population occupies a particular region, and the cell density vanishes outside of this region (Maini et al. 2004; Sengers et al. 2007), and (ii) a mathematically insightful, but less biologically realistic initial condition where the initial cell density decays exponentially as $x \rightarrow \infty$. For the biologically realistic initial conditions we always consider

6 $$\begin{aligned} u(x,0)&= 1 - H(\beta ), \end{aligned}$$

7 $$\begin{aligned} s(x,0)&= 0, \end{aligned}$$

on $0< x < \infty$, where *H*(*x*) is the usual Heaviside function and $\beta > 0$ is a constant describing the initial length of the domain that is occupied at $t=0$. For the mathematically interesting initial condition we always consider

8 $$\begin{aligned} u(x,0)&= {\left\{ \begin{array}{ll} 1, &{} \ x < \beta , \\ \text {exp}[-a(x-\beta )], &{} \ x > \beta , \end{array}\right. }\end{aligned}$$

9 $$\begin{aligned} s(x,0)&= 0, \end{aligned}$$

on $0< x < \infty$, where $a > 0$ is the decay rate. For all results we set $\beta = 10$, and we note that this choice has no impact on the long-time travelling wave solutions.

We focus on long-time numerical solutions of Eqs. (3)–(4) to explore travelling wave solutions. Details of the numerical method we use to solve the governing equations are given in Supplementary Material. Of course, the travelling wave analysis of this model is relevant on an infinite domain, but numerically we must always work with a truncated domain $0< x < X$, where *X* is chosen to be sufficiently large that the late-time numerical solutions are unaffected by the choices of *X*. All analysis corresponds to $0< x < \infty$, which is analogous to our numerical simulation domain. All algorithms required to recreate the results in this work are available on GitHub.

**Fig. 2:**
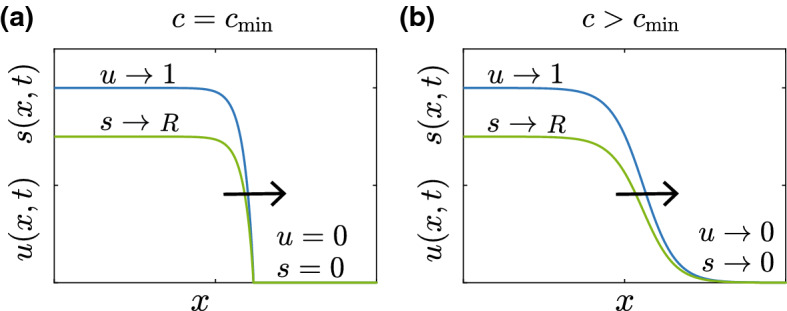
Schematic showing sharp and smooth-fronted travelling wave solutions.**a** Schematic showing a sharp-fronted travelling wave. **b** Schematic showing a smooth-fronted travelling wave. Arrows show the direction of movement (color figure online)

Before we present and discuss particular travelling wave solutions, it is convenient to state at the outset that we find the substrate invasion model leads to two types of travelling wave solutions, shown schematically in Fig. 2 where we define

10 $$\begin{aligned} R = \dfrac{r_1}{r_2}. \end{aligned}$$

The travelling wave solution in Fig. 2a arises from the biologically relevant initial conditions (6)–(7), where we see that there is a well-defined sharp front with $u=s=0$ ahead of the front, and $u \rightarrow 1^-$ and $s \rightarrow R^-$ well-behind the travelling wave front as $x \rightarrow 0^+$. Here the superscripts $+$ and − indicate a one-sided limit from above and below, respectively. In this case, as we will show, the travelling wave solution corresponds to the minimum wave speed, $c = c_{\mathrm{min}}$, which depends on the value of $r_1$ and $r_2$. In contrast, the travelling wave solution in Fig. 2b arises from the mathematically interesting initial conditions (8)–(9). In this second type of travelling wave we have the same behaviour well-behind the wave front as in Fig. 2a, since $u \rightarrow 1^-$ and $s \rightarrow R^-$ as $x \rightarrow 0^+$. However, in this case we have a smooth travelling wave with $u \rightarrow 0^+$ and $s \rightarrow 0^+$ as $x \rightarrow \infty$. Further, as we will show, these smooth-fronted travelling wave solutions move with a faster travelling wave speed, $c > c_{\mathrm{min}}$.

The fact that the substrate model gives rise to both smooth and sharp-fronted travelling wave solutions is very interesting and worthy of exploration. Throughout this work we will explore parallels between the substrate model and the Porous-Fisher model, and an obvious point of similarity is that both these models support smooth and sharp-fronted travelling wave solutions (Murray 2002; Sanchez and Maini 1994; Sherratt and Marchant 1996). As we will explore in this work, however, the differences between the smooth and sharp-fronted travelling waves in the substrate model are more subtle than the Porous-Fisher model, and we must use different methods of analysis to understand these differences.

**Fig. 3:**
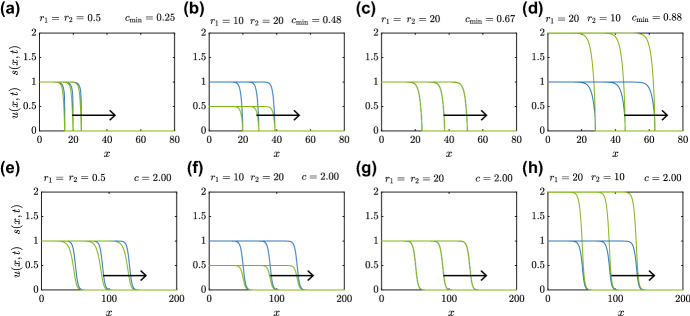
Time-dependent PDE solutions showing smooth and sharp-fronted travelling wave solutions. Sharp-fronted travelling wave solutions in (**a**)–(**d**) are obtained by solving Eqs. (3)–(5) with (6)–(7). Smooth-fronted travelling wave solutions in (**e**)–(**h**) are obtained by solving Eqs. (3)–(5) with (8)–(9) and $a=1/2$. Values of $r_1$ and $r_2$ are indicated on each subfigure, and the long-time estimate of the travelling wave speed *c* is also given to two decimal places. Each subfigure shows profiles for *u*(*x*, *t*) (blue) and *s*(*x*, *t*) (green) at $t=20, 40$ and 60, with the arrow showing the direction of increasing *t*. All numerical solutions correspond to $\Delta x = 1\times 10^{-2}$, $\Delta t = 1\times 10^{-3}$ and $\epsilon =1\times 10^{-10}$ (colour figure online)

In addition to the schematic solutions in Fig. 2, we present a range of time-dependent PDE solutions in Fig. 3 where we explore the role of varying the substrate dynamics by choosing different values of $r_1$ and $r_2$.

The results in Fig. 3a–c for the sharp-fronted travelling wave solutions show that the long-time minimum travelling wave speed, $c_{\mathrm{min}}$, depends on $r_1$ and $r_2$. In particular, comparing the results in (a)–(d) shows that $c_{\mathrm{min}}$ appears to increase with $r_1$. In contrast, the smooth-fronted travelling wave solutions in Fig. 3e–h lead to travelling wave solutions where the wave speed $c > c_{\mathrm{min}}$ appears to be independent of $r_1$ and $r_2$. These numerical solutions confirm that $s \rightarrow R^-$ as $x \rightarrow 0^-$.

**Fig. 4:**
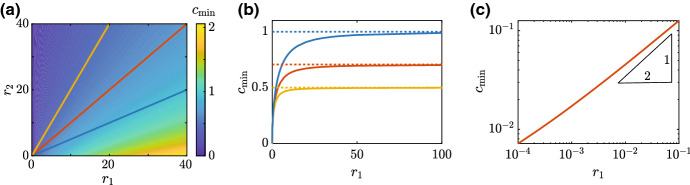
Numerical exploration of the relationship between $c_{\mathrm{min}}$, $r_1$ and $r_2$. (**a**) heat map of $c_{\mathrm{min}}$ as a function of $r_1$ and $r_2$ obtained by solving (3)–(5) with (6)–(7). The three straight lines superimposed on (**a**) correspond to $R=0.5$ (yellow), $R=1$ (red) and $R=2$ (blue), and the relationship between $c_{\mathrm{min}}$ and $r_1$ for these fixed values of *R* is given in (b), showing that $c_{\mathrm{min}} \rightarrow \sqrt{R/2}^{\,-}$ as $r_1 \rightarrow \infty$. (**c**) shows $c_{\mathrm{min}}$ as a function of $r_1$ for $R=0.5,1$ and 2, suggesting that $c_{\mathrm{min}} \sim A \sqrt{r_1}$ as $r_1 \rightarrow 0$, for some constant $A>0$. All numerical solutions correspond to $\Delta x = 1\times 10^{-2}$, $\Delta t = 1\times 10^{-2}$ and $\epsilon =1\times 10^{-10}$ (color figure online)

Now we have established that the long-time travelling wave speed for the sharp-fronted travelling wave solutions depends upon $r_1$ and $r_2$, we generate a suite of sharp-fronted travelling wave solutions numerically and estimate $c_{\mathrm{min}}$ as a function of $r_1$ and $r_2$, as reported in Fig. 4a. This heat map suggests that holding $r_2$ constant and increasing $r_1$ leads to an increase in $c_{\mathrm{min}}$. In contrast, holding $r_1$ constant and increasing $r_2$ reduces $c_{\mathrm{min}}$. To further explore this relationship we superimpose three straight lines on the heat map in Fig. 4a. These straight lines correspond to $R=0.5$ (yellow), $R=1$ (red) and $R=2$ (blue). Plotting $c_{\mathrm{min}}$ as a function of $r_1$ for these three fixed values of *R* in Fig. 4b suggest that $c_{\mathrm{min}} \rightarrow \sqrt{R/2}^{\,-}$ for fixed *R*, as $r_1 \rightarrow \infty$. As we will explain later in Sect. 2.6, this numerical observation is related to the fact that the substrate model simplifies to the Porous-Fisher model when $r_1$ and $r_2$ are sufficiently large (Buenzli et al. 2020).

The results in Fig. 4b explore the fast substrate production limit, $r_1 \rightarrow \infty$ for fixed *R*, whereas the results in Fig. 4c explore the small substrate production limit, $r_1 \rightarrow 0$. In this case we plot $c_{\mathrm{min}}$ as a function for $r_1$, for $R=0.5, 1$ and 2, and we see that the results for different values of *R* are identical, suggesting that $c_{\mathrm{min}}$ is independent of $r_2$ as $r_1 \rightarrow 0$. Furthermore, the straight line relationship on the log–log plot in Fig. 4c suggests that we have $c_{\mathrm{min}} \sim A \sqrt{r_1}$ as $r_1 \rightarrow 0$ for some constant $A>0$.

In summary, the results in Fig. 4 summarise the numerically determined relationship between $c_{\mathrm{min}}$, $r_1$, and $r_2$ for sharp-fronted travelling wave solutions of the substrate model. These numerical results are of interest because some results are consistent with well-known results for the Porous-Fisher model as we further explore in Sect. 2.6. In contrast, we also observe different behaviour that is inconsistent with the Porous-Fisher model. For example, the non-dimensional Porous-Fisher model has a positive minimum wavespeed $c_{\mathrm{min}} = 1/\sqrt{2} \approxeq 0.71$, whereas the substrate-mediated invasion model supports sharp-fronted travelling wave solutions with vanishingly small minimum wave speed, $c_{\mathrm{min}} \rightarrow 0$ as $r_1 \rightarrow 0$. Table 1 summarises the differences and similarities between travelling wave solutions of the Porous-Fisher model and the substrate model. While some of these results have only been numerically explored so far, in later sections we will provide more thorough evidence to support these numerically based observations.

**Table 1:** Key features of travelling wave solutions of the substrate-mediated invasion model with travelling wave solutions of the Porous-Fisher model

Porous-Fisher		Substrate-mediated model	
Smooth front	Sharp front	Smooth front	Sharp front
$$\begin{aligned} c = {\left\{ \begin{array}{ll} \dfrac{1}{a} &{} a < \sqrt{2}\\ \dfrac{1}{\sqrt{2}} &{} a \ge \sqrt{2}\end{array}\right. } \end{aligned}$$	$$\begin{aligned} c_{\text {min}} = {\dfrac{1}{\sqrt{2}}} \end{aligned}$$	$$\begin{aligned} c&= \dfrac{1}{a} \\ \lim _{\begin{array}{c} r_1 \rightarrow \infty \\ r_2 \rightarrow \infty \end{array}} c&= {\sqrt{\dfrac{R}{2}}}^{\ -} \end{aligned}$$	$$\begin{aligned} \lim _{r_1 \rightarrow 0^+}c_{\text {min}}&= 0^+\\ \lim _{\begin{array}{c} r_1 \rightarrow \infty \\ r_2 \rightarrow \infty \end{array}}c_{\text {min}}&= {\sqrt{\dfrac{R}{2}}}^{\ -} \end{aligned}$$

Given the numerical evidence developed in this section, we will now use phase space techniques to understand the differences between the sharp-fronted and smooth-fronted travelling wave solutions of the substrate model.

### Phase Space Analysis for Smooth Travelling Wave Solutions

In the usual way, we seek to study travelling wave solutions of Eqs. (3)–(4) by writing $u(x, t)= U(z)$ and $S(x, t)=S(z)$, where *z* is the travelling wave variable, $z=x-ct$ (Murray 2002) to give

11 $$\begin{aligned} \dfrac{\mathrm {d}}{\mathrm {d}z}\left( S \dfrac{\mathrm {d}U}{\mathrm {d}z}\right) + c\dfrac{\mathrm {d}U}{\mathrm {d}z} + U(1-U)&= 0,&-\infty< z < \infty , \end{aligned}$$

12 $$\begin{aligned} c\dfrac{\mathrm {d} S}{\mathrm {d} z} + r_1 U- r_2 S&= 0,&-\infty< z < \infty . \end{aligned}$$

Boundary conditions for the smooth travelling wave solutions are $U(z) \rightarrow 1$ and $S(z) \rightarrow R$ as $z\rightarrow -\infty$, and $U(z) \rightarrow 0$ and $S(z) \rightarrow 0$ as $z\rightarrow \infty$. Given such a smooth-fronted travelling wave solution for *U*(*z*), we can solve Eq. (12) to give

13 $$\begin{aligned} S(z)=\frac{r_1}{c}\text {exp}\left[ \dfrac{r_2 z}{c}\right] \int _{z}^{\infty } \text {exp}\left[ \dfrac{-r_2 y}{c}\right] \, U(y) \, \mathrm {d} y. \end{aligned}$$

We will make use of this result later.

Following the usual approach to studying smooth travelling wave solutions, we rewrite Eqs. (11)–(12) as a first-order system

14 $$\begin{aligned} \dfrac{\mathrm {d} U}{\mathrm {d} z}&= W, \end{aligned}$$

15 $$\begin{aligned} \dfrac{\mathrm {d} S}{\mathrm {d} z}&= -\left( \dfrac{r_1 U - r_2 S}{c}\right) , \end{aligned}$$

16 $$\begin{aligned} \dfrac{\mathrm {d} W}{\mathrm {d} z}&= W\left( \dfrac{r_1 U - r_2 S-c^2}{cS}\right) - \dfrac{ U(1-U)}{S}. \end{aligned}$$

There are two equilibrium points of the phase space: (i) $({\bar{U}},{\bar{S}},{\bar{W}}) = (1,R,0)$ as $z\rightarrow -\infty$, which corresponds to the invaded boundary; and, (ii) $({\bar{U}},{\bar{S}},{\bar{W}}) = (0,0,0)$ as $z\rightarrow \infty$, which corresponds to the uninvaded boundary.

To explore the possibility of a heteroclinic orbit connecting the two equilibrium points in the three-dimensional phase space, the Jacobian of this system is

17 $$\begin{aligned} \begin{bmatrix} 0 &{} 0 &{} 1\\ -\dfrac{r_1}{c}&{}\dfrac{r_2}{c}&{} 0\\ \displaystyle \dfrac{r_1 {\bar{W}} - c(1 - 2{\bar{U}})}{c {\bar{S}}} &{} \dfrac{(-r_1 {\bar{U}} + c^2){\bar{W}}+c{\bar{U}}(1-{\bar{U}})}{c{\bar{S}}^2}&{} \dfrac{ -r_2 {\bar{S}} +r_1 {\bar{U}} - c^2}{c{\bar{S}}}\\ \end{bmatrix}. \end{aligned}$$

We see immediately that we cannot follow the usual practice of evaluating the Jacobian at the uninvaded equilibrium point since it is not defined at $({\bar{U}},{\bar{S}},{\bar{W}}) = (0,0,0)$ and so linearisation is not useful here. In contrast, the Jacobian at the invaded equilibrium point $({\bar{U}},{\bar{S}},{\bar{W}}) = (1,R,0)$ is

18 $$\begin{aligned} \begin{bmatrix} 0 &{} 0 &{} 1\\ -\dfrac{r_1}{c}&{}\dfrac{r_2}{c}&{} 0\\ \dfrac{r_2}{r_1}&{} 0&{} -\dfrac{c r_2}{r_1}\\ \end{bmatrix}. \end{aligned}$$

The eigenvalues of this Jacobian are $\lambda _1 = r_2/c$ and $\lambda _{2,3} = (-c \pm \sqrt{c^2 + 4 R})/(2R)$. Since these eigenvalues are all real valued, with $\lambda _{1,2} > 0$ and $\lambda _3 < 0$, the invaded equilibrium point is a three-dimensional saddle point.

As just mentioned, linearisation about the uninvaded equilibrium point is not possible, and so we revisit the dynamical system (14)–(16) as $z \rightarrow \infty$ in more detail in Sect. 2.5. For now, we suppose that a smooth travelling wave *U*(*z*) decays exponentially, say

19 $$\begin{aligned} U(z) \sim C\text {exp}\left( -b z \right) \quad z \rightarrow \infty , \end{aligned}$$

where $b > 0$. Under this assumption it follows from (13) that

20 $$\begin{aligned} S(z)&\sim \dfrac{r_1}{b c + r_2}U(z), \end{aligned}$$

21 $$\begin{aligned} W(z)&\sim -b U(z), \end{aligned}$$

suggesting that *S*(*z*) and *W*(*z*) both decay to zero exponentially, at the same rate as *U*(*z*), as $z \rightarrow \infty$. Further, to leading order as $z \rightarrow \infty$, (16) gives

22 $$\begin{aligned} \dfrac{\mathrm {d} W}{\mathrm {d} z}&\sim (bc-1)\left( \dfrac{b c + r_2}{r_1}\right) \quad \text {as} \quad z \rightarrow \infty . \end{aligned}$$

At first glance this result appears inconsistent with our arguments so far, since for smooth travelling wave solutions we expect $\mathrm {d} W / \mathrm {d} z \rightarrow 0$ as $z \rightarrow \infty$, but here we have $\mathrm {d} W / \mathrm {d} z$ approaching a constant. However, by choosing $c=1/b$ we avoid this inconsistency. This choice implies that the speed of the smooth-fronted travelling wave is related to the far-field decay rate of *U*(*z*). We have tested this hypothesis numerically and found an excellent match between (19) and (21) and the shape of the smooth-fronted travelling waves for different choices of $r_1$, $r_2$ and *c*, with one example discussed in the Supplementary Material. In addition, we provide further evidence for this far-field behaviour in Sect. 2.5.

### Dispersion Relationship

We now explore how the decay rate of the initial condition, *a* in Eq. (8), affects the long-time travelling wave speed for smooth-fronted travelling wave solutions. To be consistent with our observations in Sect. 2.3, we assume that smooth-fronted travelling wave solutions for *U*(*z*) and *S*(*z*) decaying at the same rate, and we seek solutions of the form ${\tilde{u}}(x,t) \sim C \ \text {exp} \ [a(x-ct)]$ and ${\tilde{s}}(x,t) \sim D \ \text {exp} \ [a(x-ct)]$ as $x \rightarrow \infty$. Substituting these solutions into Eq. (3), and focusing on the leading edge of these solutions where ${\tilde{u}}(x,t) \ll 1$, we obtain

23 $$\begin{aligned} c = \dfrac{1}{a}, \end{aligned}$$

which relates the long-time speed of the travelling wave solution to the decay rate of the initial condition, *u*(*x*, 0).

**Fig. 5:**
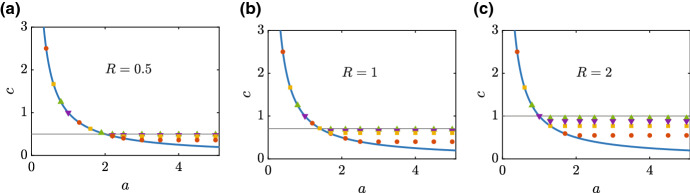
Dispersion relationship. (**a**)–(**c**) shows *c* as a function of the initial decay rate, *a*, for $R=0.5, 1$ and 2, respectively. Numerical travelling wave speeds are estimated from long-time numerical solutions of Eqs. (3)–(5) with the initial condition given by Eqs. (8)–(9) with various values of *a*. The dispersion relationship, Equation (23), is plotted (solid blue) and results for $r_1 = 1, 5, 10$ and 20 are shown in orange discs, yellow squares, purple triangles and green triangles, respectively. Each plot shows a horizontal line at $\sqrt{R/2}$, which is an upper bound for the wavespeed for large *a*. All numerical PDE solutions correspond to $\Delta x = 1\times 10^{-2}$, $\Delta t = 1\times 10^{-3}$ and $\epsilon =1\times 10^{-10}$ (color figure online)

The results in Fig. 5 explore the validity of Equation (23) by taking time-dependent PDE solutions with initial conditions (8)–(9) and varying the decay rate of *u*(*x*, 0) for various values of $r_1$ and $r_2$. In particular, we generate travelling wave solutions for $r_1 = 1, 5, 10$ and 20, for fixed $R=0.5, 1$ and 2. The results in Fig. 5a–c corresponding to $R=0.5, 1$ and 2, respectively, show that for sufficiently small *a*, we see that the long-time travelling wave speed matches Eq. (23) regardless of $r_1$ and $r_2$. These results are consistent with the initial explorations in Fig. 3e–h where we saw that the wave speed of certain smooth-fronted travelling wave solutions was independent of $r_1$ and $r_2$. As *a* increases, however, we see that *c* behaves differently. For large $a > a_{\text {crit}}$ we see that *c* approaches a constant value $c_{\text {min}}$ that is independent of *a*. Our numerical evidence suggests that this limiting constant value depends on $r_1$ and $r_2$. For completeness, on each subfigure we plot a horizontal line at $c = \sqrt{R/2}$, and we note that this value appears to be an upper-bound for *c* as *a* becomes large.

**Fig. 6:**
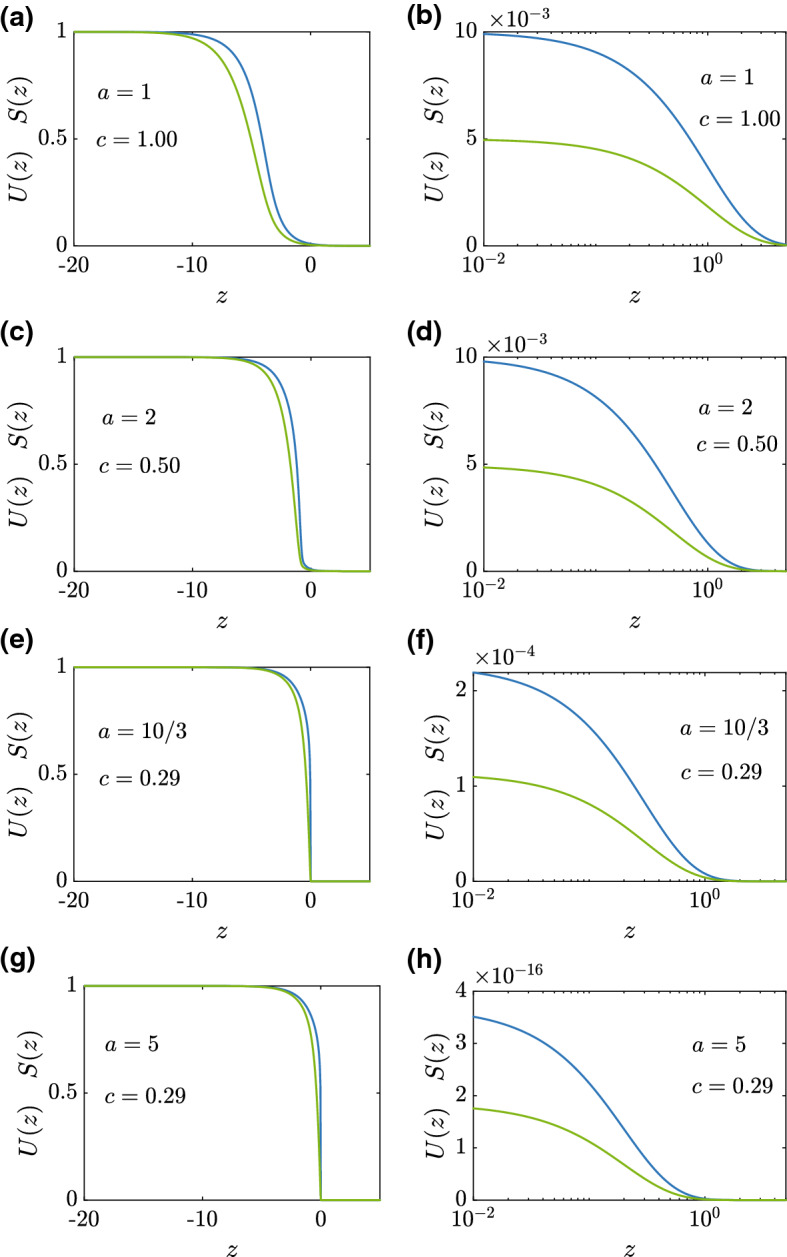
Smooth-fronted travelling wave solutions. Travelling wave solutions *U*(*z*) and *S*(*z*) are obtained by considering long-time numerical solutions of Eqs. (3)–(5) with initial conditions given by Eqs. (8)–(9) with variable decay rate, *a*. All results correspond to $r_1=r_2=1$, and results in (**a**)–(**b**), (**c**)–(**d**), (**e**)–(**f**) and (**g**)–(**h**) correspond to $a=1, 2, 10/3$ and 5, respectively, as indicated. The results in the left-most column show the various travelling wave solutions plotted on the usual scale with $0 \le U(z), S(z) \le 1$. The results in the right-most column show a magnification of the leading edge of the travelling waves (color figure online)

The transition from $c = 1/a$ for $a < a_{\text {crit}}$ to constant *c* for $a > a_{\text {crit}}$ in Fig. 5 is further explored in Fig. 6 for $r_1=r_2=1$. The long-time travelling wave solution in Fig. 6a, b evolves from an initial condition with decay rate $a=1$. This solution evolves into a smooth travelling wave with $c=1.00$, which is consistent with the dispersion relationship (23). Although it is clear that the travelling wave solution in Fig. 6a is smooth at this scale, we also plot a magnification of the leading edge of that travelling wave in Fig. 6b. We now explore a series of travelling wave solutions as *a* increases to visualise the transition reported in Fig. 5. The long-time travelling wave solution in Fig. 6c, d evolves from an initial condition with a faster decay rate, $a=2$, leading to a smooth-fronted travelling wave with $c=0.50$. Again, this result is consistent with the dispersion relationship, and the magnification of the density profiles near the leading edge in Fig. 6d confirms that the travelling wave solution is smooth. The travelling wave solution in Fig. 6e for $a=10/3$ leads to a travelling wave solution with $c=0.29$. This estimate from the long-time numerical solution of the PDE is close to the travelling wave speed predicted by the dispersion relationship. At the scale shown in Fig. 6e it might seem, at first glance, that the travelling wave is sharp, but the magnification in Fig. 6f confirms that this travelling wave is indeed smooth-fronted. Finally, the travelling wave solution in Fig. 6g for $a=5$ evolves to a travelling wave solution with $c=0.29$, which is much larger than the speed predicted by the dispersion relationship that would give $c = 1/5 = 0.2$. Again, while the travelling wave solution in Fig. 6g appears to be sharp at this scale, the magnification of the solution in Fig. 6h confirms that this solution is indeed smooth-fronted.

In summary, the dispersion relationship suggests that long-time speed of smooth-fronted travelling wave solutions is given by $c = 1/a$, where *a* is far-field the decay rate of *u*(*x*, 0). Our numerical explorations in Figs. 5, 6 confirms that this result holds for sufficiently small decay rates, $a < a_{\text {crit}}$. As the decay rate increases, $a > a_{\text {crit}}$, we observe an interesting transition for smooth-fronted travelling waves where *c* becomes independent of *a*, and greater than the speed predicted by the dispersion relationship. While these travelling wave solutions remain smooth-fronted as *a* increases, it becomes increasingly difficult to draw a visual distinction between these smooth-fronted travelling wave solutions and sharp-fronted travelling wave solutions that evolve from initial conditions with compact support, such as those travelling waves in Fig. 3a–d. We now seek to provide a geometric interpretation of the differences between these two classes of travelling wave solutions by returning to the phase space.

### Desingularised Phase Space and Slow Manifold Reduction

We now return to the phase space for travelling wave solutions and introduce a change of variables

24 $$\begin{aligned} \zeta (z) = \int _{0}^{z} \dfrac{\mathrm {d}y}{S(y)}, \end{aligned}$$

which removes the singularity in Eq. (16) when $S(z)=0$. A similar transformation to desingularise the phase plane is often used in the analysis of sharp-fronted travelling wave solutions of the Porous-Fisher model (Murray 2002). The desingularised system is given by

25 $$\begin{aligned} \dfrac{\mathrm {d} U}{\mathrm {d} \zeta }&= SW, \end{aligned}$$

26 $$\begin{aligned} \dfrac{\mathrm {d} S}{\mathrm {d} \zeta }&= -S\left( \dfrac{r_1 U - r_2 S}{c}\right) , \end{aligned}$$

27 $$\begin{aligned} \dfrac{\mathrm {d} W}{\mathrm {d} \zeta }&= W\left( \dfrac{r_1 U - r_2 S-c^2}{c}\right) - U(1-U). \end{aligned}$$

There are two equilibrium points of the desingularised phase space: (i) $({\bar{U}},{\bar{S}},{\bar{W}}) = (1,R,0)$ as $\zeta \rightarrow -\infty$, corresponding to the invaded boundary; and, (ii) $({\bar{U}},{\bar{S}},{\bar{W}}) = (0,0,0)$ as $\zeta \rightarrow \infty$, corresponding to the uninvaded boundary. It is important to point out that the phase space analysis in Sect. 2.3 was relevant only for smooth-fronted travelling wave solutions, whereas the desingularised phase space is appropriate for both the sharp-fronted and smooth-fronted travelling wave solutions. The Jacobian of this system is

28 $$\begin{aligned} \begin{bmatrix} 0 &{} {\bar{W}} &{} {\bar{S}}\\ -\dfrac{r_1 {\bar{S}}}{c}&{}\dfrac{-r_1 {\bar{U}} + 2 r_2 {\bar{S}}}{c}&{} 0\\ \dfrac{r_1 {\bar{W}} - c(1 - 2{\bar{U}})}{c} &{} -\dfrac{r_2{\bar{W}}}{c}&{} \dfrac{ -r_2 {\bar{S}} +r_1 {\bar{U}} - c^2}{c}\\ \end{bmatrix}. \end{aligned}$$

We can now consider both equilibrium points $({\bar{U}}, {\bar{S}}, {\bar{W}}) = (1,R,0)$ and $({\bar{U}}, {\bar{S}}, {\bar{W}}) = (1,0,0)$.

The Jacobian at the invaded equilibrium point, $({\bar{U}}, {\bar{S}}, {\bar{W}}) = (1,R,0)$, is

29 $$\begin{aligned} \begin{bmatrix} 0 &{} 0 &{} \dfrac{r_1}{r_2}\\ -\dfrac{r_1^2}{r_2}&{}\dfrac{r_1}{c}&{} 0\\ 1&{} 0&{} -c\\ \end{bmatrix}. \end{aligned}$$

The eigenvalues of this Jacobian are $\lambda _1 = r_1/c$ and $\lambda _{2,3} = (-c \pm \sqrt{c^2 + 4 R})/2$. Since $\lambda _{1,2} > 0$ and $\lambda _3 < 0$, the uninvaded equilibrium point is a three-dimensional saddle. These expressions are identical to the corresponding expressions in Sect. (2.3), which is not surprising since $\zeta = z$ near the invaded equilibrium point, $z \rightarrow -\infty$.

The Jacobian at the uninvaded equilibrium point, $({\bar{U}}, {\bar{S}}, {\bar{W}}) = (0,0,0)$, is

30 $$\begin{aligned} \begin{bmatrix} 0 &{} 0 &{} 0\\ 0&{}0&{} 0\\ -1&{} 0&{} -c\\ \end{bmatrix}. \end{aligned}$$

The eigenvalues are $\lambda _1 = -c$ and $\lambda _2 = \lambda _3 = 0$, which means that $({\bar{U}}, {\bar{S}}, {\bar{W}}) = (0,0,0)$ is a non-hyperbolic equilibrium point suggesting that the dynamics near this point take place on a slow manifold (Wiggins 2003). To explore these local dynamics near $({\bar{U}}, {\bar{S}}, {\bar{W}}) = (0,0,0)$ we apply the centre manifold theory to identify the slow manifold. To proceed we rotate the coordinate system using a transformation defined by the eigenvectors $[-c,0,1]^\top$, $[0,1,0]^\top$ and $[0,0,1]^\top$ that are associated with $\lambda _1$, $\lambda _2$ and $\lambda _3$, respectively. The relationship between the original unrotated coordinate system (*U*, *S*, *W*) and the rotated coordinate system $({\mathscr {U}},{\mathscr {S}},{\mathscr {W}})$ is given by the transformation (Maclaren 2020),

31 $$\begin{aligned} \begin{bmatrix} U\\ S\\ W\\ \end{bmatrix} = \begin{bmatrix} -c &{} 0 &{} 0\\ 0&{}1&{} 0\\ 1&{} 0&{} 1\\ \end{bmatrix} \begin{bmatrix} {\mathscr {U}}\\ {\mathscr {S}}\\ {\mathscr {W}}\\ \end{bmatrix}, \end{aligned}$$

and the associated inverse transformation

32 $$\begin{aligned} \begin{bmatrix} {\mathscr {U}}\\ {\mathscr {S}}\\ {\mathscr {W}}\\ \end{bmatrix} = \dfrac{1}{c} \begin{bmatrix} -1 &{} 0 &{} 0\\ 0&{}c&{} 0\\ 1&{} 0&{} c\\ \end{bmatrix} \begin{bmatrix} U\\ S\\ W\\ \end{bmatrix}. \end{aligned}$$

These transformations allow us to rewrite the dynamical system in the following format

33 $$\begin{aligned} \begin{bmatrix} \dfrac{\mathrm {d} {\mathscr {U}}}{\mathrm {d} \zeta }\\ \dfrac{\mathrm {d} {\mathscr {S}}}{\mathrm {d} \zeta }\\ \dfrac{\mathrm {d} {\mathscr {W}}}{\mathrm {d} \zeta }\\ \end{bmatrix} =&\begin{bmatrix} 0 &{} 0 &{} 0\\ 0 &{} 0 &{} 0\\ 0 &{} 0 &{} -c\\ \end{bmatrix} \begin{bmatrix} {\mathscr {U}}\\ {\mathscr {S}}\\ {\mathscr {W}}\\ \end{bmatrix} \nonumber \\&+\dfrac{1}{c} \begin{bmatrix} -\left[ {\mathscr {S}}({\mathscr {U}}+{\mathscr {W}})\right] \\ \left[ {\mathscr {S}}(r_1c{\mathscr {U}}+r_2{\mathscr {S}})\right] \\ \left[ ({\mathscr {U}}+{\mathscr {W}})\left[ -r_1c{\mathscr {U}} + (1-r_2){\mathscr {S}}\right] + c^2{\mathscr {U}}(1+c{\mathscr {U}})\right] \\ \end{bmatrix}. \end{aligned}$$

To find the slow manifold we take the usual approach of writing the fast dynamics associated with $\lambda _1$ as a function of the slow dynamics that are associated with the zero eigenvalues by assuming that slow manifold can be locally expressed as a quadratic in ${\mathscr {U}}$ and ${\mathscr {V}}$. Equating coefficients with the tangency condition (Wiggins 2003) gives the slow manifold,

34 $$\begin{aligned} {\mathscr {W}}({\mathscr {U}},{\mathscr {S}})=\dfrac{1}{c^2}\left[ c(c^2-r_1) {\mathscr {U}}^2 + (1-r_2) {\mathscr {U}}{\mathscr {S}}\right] , \end{aligned}$$

and the dynamics on the slow manifold are given by

35 $$\begin{aligned} \dfrac{\mathrm {d} {\mathscr {U}}}{\mathrm {d} \zeta }&=-\dfrac{1}{c^3}\left[ c(c^2-r_1){\mathscr {U}}^2{\mathscr {S}}+(1-r_2){\mathscr {U}}{\mathscr {S}}^2+c^2{\mathscr {S}}{\mathscr {U}}\right] , \end{aligned}$$

36 $$\begin{aligned} \dfrac{\mathrm {d} {\mathscr {S}}}{\mathrm {d} \zeta }&=\dfrac{1}{c}\left[ r_2{\mathscr {S}}^2+r_1c{\mathscr {U}}{\mathscr {S}}\right] . \end{aligned}$$

We can now rewrite the slow manifold and the dynamics on the slow manifold in the original, unrotated coordinate system, giving

37 $$\begin{aligned} W(U,S)=\dfrac{1}{c^3}\left[ (c^2-r_1)U^2 - (1-r_2) U S - c^2 U\right] , \end{aligned}$$

and

38 $$\begin{aligned} \dfrac{\mathrm {d} U}{\mathrm {d} \zeta }&=\dfrac{1}{c^3}\left[ (c^2-r_1)SU^2 - (1-r_2) U S^2 - c^2SU\right] , \end{aligned}$$

39 $$\begin{aligned} \dfrac{\mathrm {d} S}{\mathrm {d} \zeta }&=\dfrac{1}{c} \left[ r_2 S^2 - r_1 U S\right] , \end{aligned}$$

which could alternatively be derived using mathematical software (Roberts 2015). The advantage of the coordinate transformation method is the process is quite transparent and geometrically intuitive, whereas the advantage of the alternative approach is that it avoids the change of basis. Regardless, we arrive at the same result using either approach.

**Fig. 7:**
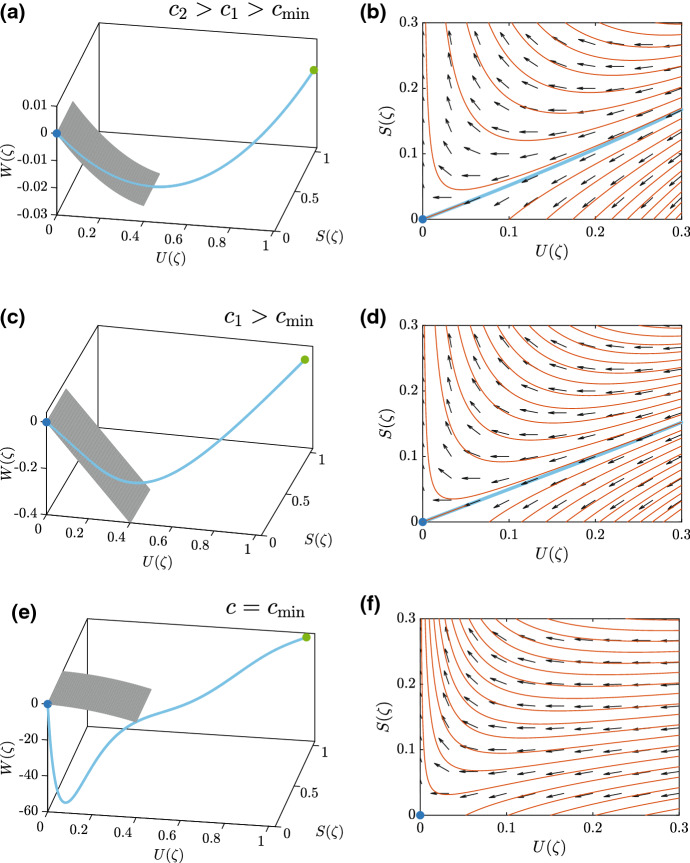
Desingularised phase space and slow manifold reduction. All results correspond to $r_1=r_2=1$. Results in: (**a**)–(**b**) correspond to a smooth-fronted travelling wave with $c_2=10$; (**c**)–(**d**) correspond to a smooth-fronted travelling wave with $c_1=1$; and, (**e**)–(**f**) correspond to a sharp-fronted travelling wave with $c_{\mathrm{min}} = 0.29$. The results in the left-most column show the three-dimensional desingularised phase space with the invaded equilibrium point (green dot), the uninvaded equilibrium point (blue dot) and the slow manifold (grey surface). The results in the right-most column show the vector field on the slow manifold, superimposed with several solution trajectories, including the heteroclinic orbit (blue) and several unphysical trajectories (red). The heteroclinic orbit is obtained by solving Eqs. (3)–(5) numerically with appropriate initial conditions. For (**a**)–(**b**) and (**c**)–(**d**) the initial conditions are given by Eqs. (8)–(9) with $a=1/10$ and $a=1$, respectively. For (**e**)–(**f**) the initial conditions are given by Eqs. (6)–(7). All numerical PDE solutions correspond to $\Delta x = 1\times 10^{-4}$, $\Delta t = 1\times 10^{-3}$ and $\epsilon =1\times 10^{-4}$ (color figure online)

With these tools we may now plot the phase space including the two equilibrium points, and superimpose the slow manifold and the heteroclinic orbit obtained be rewriting the long-time PDE solution in terms of the $(U(\zeta ), S(\zeta ), W(\zeta ))$ coordinates. This information is summarised in Fig. 7 for two smooth-fronted travelling waves and one sharp-fronted travelling wave, each with $r_1=r_2=1$. Before considering Fig. 7 in detail, note that a small *S* and *U* analysis of (38)–(39) shows that the heteroclinic orbit must have $U \sim (r_2+1)S/r_1$ as $S \rightarrow 0^+$, meaning that the slope of the heteroclinic orbit is $r_1/(r_2+1)$ in the *US*-plane near the origin, and $U \sim A \text {exp}(-z/c)$ and $S \sim B \text {exp}(-z/c)$, for some constants $A>0$, $B>0$, as $z \rightarrow \infty$ for smooth-fronted travelling wave solutions. These results for the flow on the slow manifold confirm (19)–(20) with $c=1/b$.

Figure 7a shows the three-dimensional desingularised phase space together with the invaded equilibrium point in green, the uninvaded equilibrium point in blue, the heteroclinic orbit in solid blue and the slow manifold in grey. In this case we have $c_2 = 10$ and we see that, as expected, the heteroclinic orbit enters the uninvaded equilibrium point after moving along the slow manifold. In Fig. 7b we plot the slow manifold locally around the uninvaded equilibrium point together with the vector field defined by Eqs. (38)–(39). The heteroclinic orbit from the long-time PDE solution is shown in blue. We see that the heteroclinic orbit is tangential to the vector field and enters the uninvaded equilibrium point. For completeness we also solve Eqs. (38)–(39) numerically to show a number of other solution trajectories on the slow manifold in red. While these other solution curves are valid solutions of Eqs. (38)–(39), they are unphysical in the sense that they are not associated with the travelling wave solution since they do not form a heteroclinic orbit joining the invaded and uninvaded equilibrium points. Figure 7b, c shows a similar set of results to those in Fig. 7a, b for a different smooth-fronted travelling wave, this time with $c_1 = 1$. Again we see that the heteroclinic orbit moves into the uninvaded equilibrium point along the slow manifold in Fig. 7c, with additional details shown on the slow manifold in Fig. 7d. Interestingly, the results in Fig. 7e, f, for a sharp-fronted travelling wave with $c_{\mathrm{min}} = 0.29$ are quite different to the smooth-fronted travelling waves in Fig. 7a–d. Here the heteroclinic orbit joining the invaded and uninvaded equilibrium points enters the uninvaded equilibrium point directly, without moving along the slow manifold. This difference is highlighted in Fig. 7d where we see that there is no component of the heteroclinic orbit on the slow manifold. These results in Fig. 7 are for one particular choice of $r_1=r_2=1$, and similar results for different choices of $r_1$ and $r_2$ show the same qualitative behaviour (Supplementary Material).

**Fig. 8:**
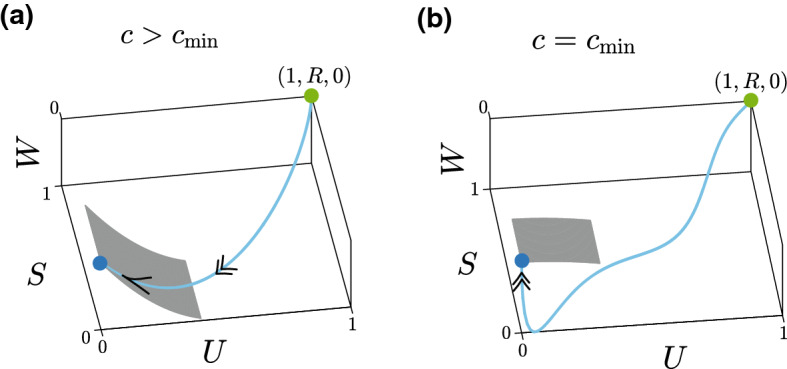
Schematic distinction between smooth-fronted and sharp-fronted travelling wave solutions. The schematic in **a** corresponds to a smooth-fronted travelling wave solution with $c > c_{\mathrm{min}}$, where the heteroclinic orbit (blue) in the desingularised phase space moves into the (0, 0, 0) along the slow manifold (grey surface). The schematic in **b** corresponds to a sharp-fronted travelling wave solution with $c = c_{\mathrm{min}}$, where the heteroclinic orbit (blue) enters the uninvaded equilibrium point, (0, 0, 0), without moving along the slow manifold (grey surface) (color figure online)

In summary, these results show us that we can make a simple geometric distinction between smooth-fronted travelling waves and sharp-fronted travelling waves using the slow manifold reduction. Smooth-fronted travelling waves involve a heteroclinic orbit joining $({\bar{U}},{\bar{S}},{\bar{W}}) = (1,R,0)$ and $({\bar{U}},{\bar{S}},{\bar{W}}) = (0,0,0)$, such that the heteroclinic orbit enters (0, 0, 0) along the slow manifold, given by Eq. (37). In contrast, sharp-fronted travelling waves involve a heteroclinic orbit joining the same two equilibrium points, with the difference being that the heteroclinic orbit enters (0, 0, 0) directly, without moving along the slow manifold. These differences are summarised schematically in Fig. 8.

It is worth noting that the computational phase space tools in Figure 7a, c and e provide physical insight into the interpretation of the minimum wave speed, $$c_{\text {min}}$$, for the substrate model. While it is not possible to compute a long-time PDE solution with $$c < c_{\text {min}}$$, it is straightforward to plot the three-dimensional phase space and integrate Eqs. (25)-(27) numerically to explore various trajectories in the relevant octant where $$U \ge 0$$, $$S \ge 0$$ and $$W \le 0$$. These explorations show that we can identity a unique trajectory that enters the origin just like we did for $$c \ge c_{\text {min}}$$, however part of this trajectory has $$U < 0$$ which is why it can never be associated with a physically relevant travelling wave solutions (Supplementary Material). This observation shares similarities and differences with the phase plane analysis of the classical Fisher-KPP model, where the exact result $$c_{\text {min}} = 2$$ is found by ensuring that $$U > 0$$ near the origin (Murray 2002). In the simpler Fisher-KPP model, the origin is an equilibrium point and so linearisation gives us the local properties of the phase plane, leading to this result. Similar methodology applies for more complicated generalisations of the Fisher-KPP model (Vittadello et al. 2018). In the case of our substrate model, it appears that $$c_{\text {min}}$$ is also defined by requiring that $$U > 0 $$ along the heteroclinic orbit (Supplementary Material). Conversely, numerical explorations show that when $$c < c_{\text {min}}$$ we observe that $$U < 0$$ for portions of the orbit that do not pass through a neighbourhood of the equilibrium point. This observation suggests that linearisation cannot be used to find a mathematical expression for $$c_{\text {min}}$$.

### Approximate Solution for Sharp-Fronted Travelling Waves

For the next part of this work we further develop an understanding of how the shape of the travelling wave profiles depends upon the parameters in the mathematical model. We will derive two approximations: one for sharp-fronted travelling wave solutions, and the other for smooth-fronted travelling wave solutions. In both cases we test our approximations using full time-dependent PDE solutions. These results are both inherently mathematically interesting as well as being of practical value because relating properties of the travelling wave solutions, such as their speed and shape, to the parameters in the mathematical model is useful if we consider estimating parameters to match experimental observations.

The numerical results in Sect. 2.2 imply a relationship between the substrate model and the Porous-Fisher model, which we now explore further. For fast substrate production and decay, $r_1\gg 1$ and $r_2\gg 1$, respectively, we anticipate that Eq. (4) gives approximately $s = Ru$, and that Eq. (3) is approximately

40 $$\begin{aligned} \dfrac{\partial u}{\partial t}= R\dfrac{\partial }{\partial x}\left( u \dfrac{\partial u}{\partial x}\right) + u(1-u), \quad 0< x < \infty , \end{aligned}$$

which is the non-dimensional Porous-Fisher model with the diffusion term scaled by the constant *R*. Therefore, we can make use of known results for the Porous-Fisher model in this limit. In particular, sharp-fronted travelling wave solutions of the Porous-Fisher model are known to have the closed-form solution (Murray 2002; Sherratt and Marchant 1996)

41 $$\begin{aligned} U(z)&= {\left\{ \begin{array}{ll} 1-\text {exp}\left( \dfrac{z-z_c}{2c}\right) , &{} z<z_c,\\ 0, &{} z > z_c, \end{array}\right. } \end{aligned}$$

42 $$\begin{aligned} S(z)&= R U(z) \quad \quad \, \, \, -\infty< z < \infty , \end{aligned}$$

where $c = c_{\mathrm{min}} = \sqrt{R/2}$ and $z_c$ is the location of the sharp front (Murray 2002). Note that Eq. (42) is equivalent to substituting (41) into (13) and evaluating the resulting expression in the limit that $r_1 \rightarrow \infty$ and $r_2 \rightarrow \infty$.

**Fig. 9:**
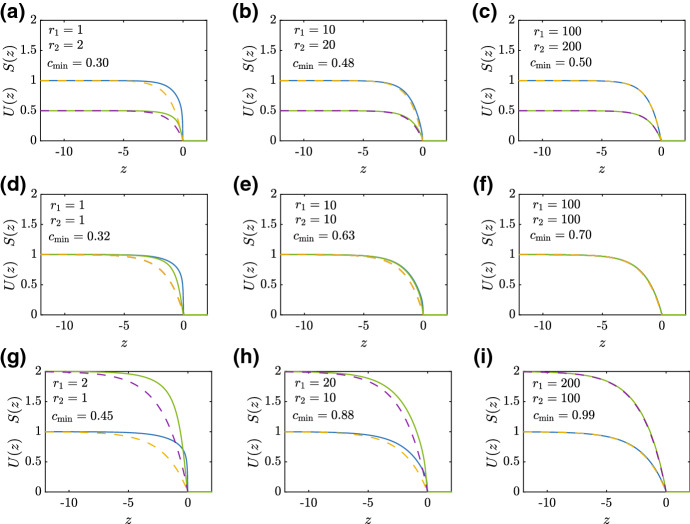
Approximate shape of sharp-fronted travelling wave solutions. Various numerical travelling wave solutions, *U*(*z*) and *S*(*z*), obtained by solving Eqs. (3)–(7) are compared with the approximation given by Equations (41)–(42), where *z* is shifted so that $z_c=0$. Results in (**a**)–(**c**), (**d**)–(**f**) and (**g**)–(**i**) correspond to $R=0.5, 1$ and 2, respectively. Each subfigure shows the appropriate value of $r_1$, $r_2$ and $c_{\mathrm{min}}$. All numerical PDE solutions correspond to $\Delta x = 1\times 10^{-2}$, $\Delta t = 1\times 10^{-3}$ and $\epsilon =1\times 10^{-10}$ (color figure online)

The results in Fig. 9 examine how late-time numerical PDE solutions can be approximated by Eqs. (41)–(42). Results in (a)–(c), (d)–(f) and (g)–(i) correspond to $R=0.5, 1$ and 2, respectively, and in each case we see that Equations (41)–(42) provide a good match with the shape of the travelling wave solution of the substrate model as $r_1$ and $r_2$ increase.

### Approximate Solution for Smooth-Fronted Travelling Waves

Previous results in Fig. 8 suggest that smooth-fronted travelling waves become less steep as *c* increases, implying that $W(z)=\mathrm {d}U / \mathrm {d}z \rightarrow 0$ as $c \rightarrow \infty$. Following the work of Canosa we make use of this observation to develop a perturbation solution by re-scaling the independent variable, ${\hat{z}} = z/c$ to give (Canosa 1973),

43 $$\begin{aligned} \dfrac{1}{c^2}\dfrac{\mathrm {d}}{\mathrm {d}{\hat{z}}}\left( S \dfrac{\mathrm {d}U}{\mathrm {d}{\hat{z}}}\right) + \dfrac{\mathrm {d}U}{\mathrm {d}{\hat{z}}} + U(1-U)&= 0,&-\infty< {\hat{z}} < \infty , \end{aligned}$$

44 $$\begin{aligned} \dfrac{\mathrm {d} S}{\mathrm {d} {\hat{z}}} + r_1 U- r_2 S&= 0,&-\infty< {\hat{z}} < \infty . \end{aligned}$$

To proceed, we seek a perturbation solution in terms of the small parameter $1/c^2$ by expanding the dependent variables in a power series (Murray 1984),

45 $$\begin{aligned} U({\hat{z}}) = \sum _{n=0}^{\infty }c^{-2n} U_n({\hat{z}}), \quad S({\hat{z}}) = \sum _{n=0}^{\infty }c^{-2n} S_n({\hat{z}}). \end{aligned}$$

Substituting these power series into Eqs. (43)–(44) and truncating after the first few terms gives

46 $$\begin{aligned}&\dfrac{\mathrm {d}U_0}{\mathrm {d}{\hat{z}}} + U_0(1-U_0) = 0, \end{aligned}$$

47 $$\begin{aligned}&\dfrac{\mathrm {d} S_0}{\mathrm {d} {\hat{z}}} + r_1 U_0 - r_2 S_0 = 0 , \end{aligned}$$

48 $$\begin{aligned}&\dfrac{\mathrm {d} }{\mathrm {d}{\hat{z}}}\left( S_0\dfrac{\mathrm {d} U_0}{\mathrm {d}{\hat{z}}}\right) + \dfrac{\mathrm {d} U_1}{\mathrm {d}{\hat{z}}} + U_1(1-2U_0)= 0, \end{aligned}$$

with boundary conditions $U_0 \rightarrow 1$, $U_1 \rightarrow 0$ and $S_0 \rightarrow R$ as ${\hat{z}}\rightarrow -\infty$, and $U_0 \rightarrow 0$, $U_1 \rightarrow 0$ and $S_0 \rightarrow 0$ as ${\hat{z}}\rightarrow \infty$. It is straightforward to solve these differential equations for $U_0({\hat{z}})$, $U_1({\hat{z}})$ and $S_0({\hat{z}})$; however, additional terms in the perturbation solution are governed by differential equations that do not have closed-form solutions. Regardless, as we shall now show, these first few terms in the perturbation solution provide accurate approximations, even for relatively small values of *c*.

The solution of Eq. (46) is

49 $$\begin{aligned} U_0(z) = \dfrac{1}{1+\text {exp}\left( {\hat{z}}\right) }, \end{aligned}$$

where we have arbitrarily chosen the integration constant so that $U_0(0)=1/2$. Given $U_0(z)$, we solve (47) using an integrating factor to give

50 $$\begin{aligned} S_0({\hat{z}}) = -r_1 \text {exp}\left( r_2 {\hat{z}}\right) \int _{{\hat{z}}}^{\infty }\dfrac{\text {exp}\left( -r_2 {\hat{z}}\right) }{1 + \text {exp}\left( {\hat{z}}\right) } \, \mathrm {d}{\hat{z}}. \end{aligned}$$

If $r_2$ is an integer we obtain

51 $$\begin{aligned} S_0({\hat{z}}) = (-1)^{r_2}\text {exp}\left( r_2 {\hat{z}}\right) r_1\left[ \ln \left( \text {exp}\left[ -{\hat{z}}\right] +1\right) +\sum _{n=1}^{r_2}\dfrac{\text {exp}\left( -n {\hat{z}}\right) }{n\left( -1\right) ^n}\right] . \end{aligned}$$

If $r_2$ is not an integer there is no closed-form expression for $S_0({\hat{z}})$ that we could find. For particular integer choices of $r_1$ the expression for $S_0({\hat{z}})$ is quite simple. For example, with $r_2=1$ we have $S_0({\hat{z}}) = r_1\left[ 1-\text {exp}\left( {\hat{z}}\right) \ln (\text {exp}\left[ -{\hat{z}}\right] +1)\right]$, whereas for $r_2=2$ we have $S_0({\hat{z}}) =r_1\left[ 1/2-\text {exp}\left( {\hat{z}}\right) +\text {exp}\left( 2{\hat{z}}\right) \ln (\text {exp}\left[ -{\hat{z}}\right] +1)\right]$. The solution for $U_1({\hat{z}})$ is obtained by integrating Eq. (48) using an integrating factor to give

52 $$\begin{aligned} U_1({\hat{z}}) = \dfrac{\text {exp}\left( {\hat{z}}\right) }{(1 + \text {exp}\left[ {\hat{z}}\right] )^2}\int _{{\hat{z}}}^{\infty } \dfrac{\mathrm {d} }{\mathrm {d}{\hat{z}}}\left[ S_0\dfrac{\text {exp}\left( {\hat{z}}\right) }{(1+\text {exp}\left[ {\hat{z}}\right] )^2}\right] \left[ \dfrac{(1 + \text {exp}\left[ {\hat{z}}\right] )^2 }{ \text {exp}\left( {\hat{z}}\right) }\right] \mathrm {d}{\hat{z}}. \qquad \end{aligned}$$

Since this expression for $U_1({\hat{z}})$ depends upon the expression for $S_0({\hat{z}})$, we can only obtain closed-form expressions for $U_1({\hat{z}})$ for integer values of $r_2$. In these cases, expressions for $U_1({\hat{z}})$ are relatively complicated and so we include these expressions in Supplementary Material. We note that care is required when evaluating $U_1({\hat{z}})$ since the expression is indeterminate for large ${\hat{z}}$. We address this simply by expanding $U_1({\hat{z}})$ in a Taylor series as ${\hat{z}} \rightarrow \infty$ and plotting the series expansion for large ${\hat{z}}$.

**Fig. 10:**
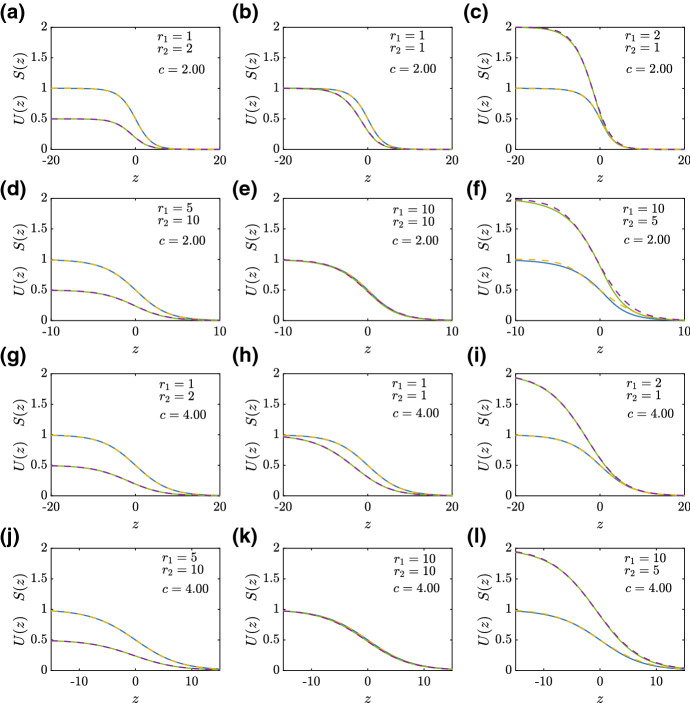
Approximate shape of smooth-fronted travelling wave solutions. Results in (**a**)–(**f**) and (**g**)–(**l**) compare the numerical and perturbation solutions for $c=2.00$ and $c=4.00$, respectively. The results in the left-most column correspond to $R=0.5$, those in the central column correspond to $R=1$, and those in the right-most column correspond to $R=2$. Numerical solutions correspond to late-time numerical solutions of Eqs. (3)–(5), with initial conditions given by Eqs. (8)–(9) with appropriate values of *a*. Numerical solutions of *U*(*z*) and *S*(*z*) are shown in blue and green, respectively. Each numerical solution is superimposed with an ${\mathcal {O}}(1)$ perturbation solution for *S*(*z*) and an ${\mathcal {O}}(1/c^2)$ for *U*(*z*), and these perturbation solutions are shown in yellow and purple dashed curves, respectively (color figure online)

Results in Fig. 10 compare the shapes of various smooth-fronted travelling wave solutions, for $c=2$ and $c=4$, with the ${\mathcal {O}}(1)$ perturbation solution for *S*(*z*) and the ${\mathcal {O}}(c^{-2})$ perturbation solution for *U*(*z*). These comparisons are made across a range of values of $r_1$ and $r_2$, and for $c=4$ the perturbation solutions are indistinguishable from the late-time numerical solutions. In cases where $c=2$ we begin to see a small departure between the numerical and perturbation approximations. Given that the perturbation solutions are valid in the limit $c \rightarrow \infty$, the quality of match in Fig. 10 for $c=2$ and $c=4$ is quite good.

## Conclusion and Future Work

In this study we investigated a minimal model of cell invasion that couples cell migration, cell proliferation and cell substrate production and decay. A key feature of the mathematical model is that the diffusive flux is proportional to the substrate density so that the flux vanishes when the substrate is absent. This feature leads to predictions of tissue formation involving the propagation of well-defined sharp fronts, and two-dimensional numerical simulations of the mathematical model recapitulate key features of recent experiments that involved the formation of thin tissues grown on 3D-printed scaffolds (Lanaro et al. 2021). To gain a deeper understanding of how the rate of substrate production and decay affects the rate of tissue production, the focus of this work is to study solutions of the substrate model in a one-dimensional geometry.

Preliminary numerical simulations of the substrate model in one dimension indicate that the mathematical model supports two types of travelling wave solutions. As we show, sharp-fronted travelling waves that propagate with a minimum wave speed, $c_{\mathrm{min}}$, evolve from initial conditions with compact support, whereas smooth-fronted travelling waves that move with a faster wave speeds, $c > c_{\mathrm{min}}$, evolve from initial conditions where the density decays exponentially with position. These numerical features are reminiscent of established features of travelling wave solutions of the well-known Porous-Fisher model.

Much of our analysis focuses on exploring the relationships between smooth-fronted and sharp-fronted travelling wave solutions, and here key features of the analysis of the substrate model are very different to the analysis of the Porous-Fisher model. For example, there are three equilibrium points in the desingularised phase plane for the Porous-Fisher model whereby smooth-fronted travelling wave solutions are characterised by a heteroclinic orbit that enters $({\bar{U}},{\bar{V}}) = (0,0)$, whereas sharp-fronted travelling wave solutions involves a heteroclinic orbit that enters $({\bar{U}},{\bar{V}}) = (0,-c)$. In contrast, the desingularised phase space for the substrate model involves two equilibrium points only. This means that both smooth-fronted and sharp-fronted travelling waves correspond to heteroclinic orbits that enter $({\bar{U}}, {\bar{S}}, {\bar{W}}) = (0,0,0)$, which is fundamentally different to the Porous-Fisher model. We provide a geometric interpretation that explains the difference between sharp-fronted and smooth-fronted travelling wave solutions since smooth-fronted travelling wave solutions are associated with a heteroclinic orbit that enters the origin in the desingularised phase space by moving along a slow manifold. In contrast, sharp-fronted travelling wave solutions are associated with a heteroclinic orbit that enters the origin of the desingularised phase space directly, without moving along the slow manifold. Additionally, we also develop and test useful closed-form expressions that describe the shape of the travelling wave solutions in various limits. In particular, we provide accurate approximations for the shape of sharp-fronted travelling waves for sufficiently large $r_1$ and $r_2$, as well as accurate approximation of the shape of the smooth-fronted travelling wave solutions relevant for large *c*.

There are many avenues for extending the current work, and these options include further analysis of the current model as well as conducting parallel analysis for related mathematical models. In terms of the current model, our analysis has not provided any relationship between $c_{\mathrm{min}}$ and the two parameters in the nondimensional model, $r_1$ and $r_2$. For simpler mathematical models, such as the Fisher-KPP model, the relationship between the minimum wave speed and the parameters in the model arises by linearising about the leading edge of the travelling wave (Murray 2002). As we have shown, an interesting feature of the substrate model is that standard techniques to linearise about the leading edge do not apply. Another possibility for extending the analysis of this model would be to consider the mathematical model in two-dimensions, such as describing the late-time dynamics of hole-closing phenomena (McCue et al. 2019).

A different class of extensions of this work would be to consider generalising the nonlinear diffusion term in the substrate model, such as

53 $$\begin{aligned}&\dfrac{\partial u}{\partial t}= \dfrac{\partial }{\partial x}\left( {\mathcal {D}}(s) \dfrac{\partial u}{\partial x}\right) + u(1-u),&0< x < \infty \end{aligned}$$

54 $$\begin{aligned}&\dfrac{\partial s}{\partial t}= r_1 u - r_2 s,&0< x < \infty . \end{aligned}$$

This generalised substrate model involves a nonlinear diffusivity function, ${\mathcal {D}}(s)$. We anticipate that nonlinear diffusivity functions with the property ${\mathcal {D}}(0) = 0$ will support sharp-fronted travelling wave solutions, and there are many such candidate functions. One option of interest is a power-law diffusivity ${\mathcal {D}}(s) = s^n$, where *n* is some exponent. It would be interesting to explore how different choices of *n* affect various qualitative and quantitative features of the travelling wave solutions that have been established in the present study for $n=1$. Other options for generalising the model include considering nonlinear source terms and/or an additional linear diffusion term in Eq. (54). Preliminary numerical investigations suggest that both these generalisations lead to travelling wave solutions; however, introducing a linear diffusion term for *s* means that the travelling wave solutions are always smooth-fronted. We will return to analyse these extensions more thoroughly in the future.

## Supplementary Information

Below is the link to the electronic supplementary material.

Supplementary file 1 (pdf 3709 KB)

## Data Availability

All algorithms and software required to reproduce the results in this work are available on GitHub.
