# Supplementary material for: A Continuum Mathematical Model of Substrate-Mediated Tissue Growth
Source: Bull Math Biol. 2022 Mar 2;84(4):49. doi: 10.1007/s11538-022-01005-7 (PMC8891221; doi:10.1007/s11538-022-01005-7)
Supplement: Supplementary file 1 — (pdf 3709 KB) [file 11538_2022_1005_MOESM1_ESM.pdf]

Noname manuscript No.
(will be inserted by the editor)

Supplementary Material

Maud El-Hachem[1] · **Scott W McCue**[1] ·
Matthew J Simpson[1*]

Received: date / Accepted: date

[1] School of Mathematical Sciences, Queensland University of Technology, Brisbane, Australia
E-mail: matthew.simpson@qut.edu.au

S1 Numerical methods

S1.1 Two–Dimensional partial differential equations

Numerical solutions of the substrate model in two–dimensions are obtained by re-writing Equations (1)–(2) as

$$\frac{\partial u}{\partial t} = \frac{\partial}{\partial x}\left[\mathcal{D}(s)\frac{\partial u}{\partial x}\right] + \frac{\partial}{\partial y}\left[\mathcal{D}(s)\frac{\partial u}{\partial y}\right] + f(u), \tag{S1}$$

$$\frac{\partial s}{\partial t} = g(u, s), \tag{S2}$$

where we have written the mathematical model in terms of a general nonlinear diffusivity function $\mathcal{D}(s)$, and general source terms, $f(u)$ and $g(u, s)$. To be consistent with our dimensional model we have $\mathcal{D}(s) = Ds/K_s$, $f(u) = \lambda u(1 - u/K_u)$ and $g(u, s) = r_1 u - r_2 s$, but our numerical method can deal with other functional forms if required. Our aim is to obtain numerical solutions of Equations (S1)–(S2) on the square domain $\Omega = \{(x, y), 0 < x < L, 0 < y < L\}$. For convenience we assume that the origin is at the lower left corner of the domain and we discretise Ω on a spatially uniform finite difference mesh with mesh spacing $\Delta x = \Delta y > 0$. We index the mesh in the usual way so that the coordinates of each mesh point are (x_i, y_j), with $i = 0, 1, 2, \ldots, I$ and $j = 0, 1, 2, \ldots, J$. Since we always consider a square mesh we have $I = J$. All numerical results correspond to a 101×101 mesh which, with $L = 300$ μm, gives $\Delta x = 3$ μm. We found that solutions obtained on a finer mesh gave visually indistinguishable results for the parameter values that we considered.

We solve Equations (S1)–(S2) using a standard method of lines approach so that at each internal mesh point we have

$$\begin{aligned}\frac{\mathrm{d}u_{i,j}}{\mathrm{d}t} &= \frac{1}{2\Delta x^2}\left[\left(\mathcal{D}(s_{i,j}) + \mathcal{D}(s_{i+1,j})\right)\left(u_{i+1,j} - u_{i,j}\right)\right. \\ &\quad \left. - \left(\mathcal{D}(s_{i,j}) + \mathcal{D}(s_{i-1,j})\right)\left(u_{i,j} - u_{i-1,j}\right)\right] \\ &\quad + \frac{1}{2\Delta x^2}\left[\left(\mathcal{D}(s_{i,j}) + \mathcal{D}(s_{i,j+1})\right)\left(u_{i,j+1} - u_{i,j}\right)\right. \\ &\quad \left. - \left(\mathcal{D}(s_{i,j}) + \mathcal{D}(s_{i,j-1})\right)\left(u_{i,j} - u_{i,j-1}\right)\right] + f(u_{i,j}), \qquad \text{(S3)} \\ \frac{\mathrm{d}s_{i,j}}{\mathrm{d}t} &= g(u_{i,j}, s_{i,j}), \qquad \text{(S4)}\end{aligned}$$

where we have approximated the internode diffusivity with an arithmetic average. These discretised equations are valid at central nodes, $i = 1, 2, \ldots, I-1$ and $j = 1, 2, \ldots, I-1$, and we implement Dirichlet boundary conditions for both dependent variables along all boundaries. We explored various temporal integration methods and found that all standard approaches led to grid–independent results when Δx and the temporal step size are sufficiently small. For simplicity all results in the main document correspond to the simplest forward Euler temporal integration with constant time steps of duration Δt.

S1.2 One–dimensional partial differential equations

To solve Equations (3)–(4) we consider a domain $0 < x < L$ that we discretise into m equally–sized intervals with spacing Δx. We approximate Equations (3)–(4) using a central difference approximation for the spatial derivative. Since we use this algorithm to study long–time travelling wave solutions we approximate

the temporal derivative with an implicit Euler approximation, giving

$$\frac{u_i^{j+1}-u_i^j}{\Delta t} = \frac{1}{2\Delta x^2}\left[(s_{i+1}^{j+1}+s_i^{j+1})(u_{i+1}^{j+1}-u_i^{j+1})-(s_i^{j+1}+s_{i-1}^{j+1})(u_i^{j+1}-u_{i-1}^{j+1})\right] + u_i^{j+1}\left(1-u_i^{j+1}\right), \tag{S5}$$

$$\frac{s_i^{j+1}-s_i^j}{\Delta t} = r_1 u_i^{j+1} - r_2 s_i^{j+1}, \tag{S6}$$

for $i = 2, \ldots, m-1$, where $m = 1/h + 1$ is the total number of spatial nodes on the finite difference mesh, and the index j represents the time index so that $u_i^j \approx u(x,t)$ and $s_i^j \approx s(x,t)$, where $x = (i-1)\Delta x$ and $t = j\Delta t$. The boundary conditions for u are discretized to give

$$u_2^{j+1} - u_1^{j+1} = 0, \quad u_m^{j+1} = 0. \tag{S7}$$

We solve the resulting system of nonlinear algebraic equations for u using Newton-Raphson algorithm with convergence tolerance ϵ. Once we have the updated solutions for u_i^{j+1}, updated estimates of s_i^{j+1} are given by Equation (S6). For all results presented in the main document we carefully checked that re-computing the solutions with smaller Δt, Δx and ϵ gave visually indistinguishable results for the parameter values that we considered.

S1.3 Numerical estimate of the travelling wave speed c

We estimate the travelling wave speed by specifying a particular contour value, $u(x,t) = u^*$ and use linear interpolation to estimate x^* such that $u(x^*,t) = u^*$ at each time step. With this data we then calculate

$$c = \frac{x^*(t+\Delta t) - x^*(t)}{\Delta t}, \tag{S8}$$

at each time step, which we find settles to a constant value for sufficiently large t. Given this time series of estimates for c we fit a straight line to the late–time data

to provide an estimate of c. All results in this work correspond to $u^* = 0.5$, but we find that our results are insensitive to this choice and other values of $u^* \in (0, 1)$ give the same results provided that δt and h are chosen to be sufficiently small.

S1.4 Phase plane on the slow manifold

We solve Equations (38)–(39) numerically to estimate trajectories on the slow manifold using Heun's method with a constant step size dζ. The vector field of the dynamical system is plotted on the phase planes using the MATLAB *quiver* function (Mathworks 2021).

S2 Additional results and discussion

S2.1 Far–field behaviour of smooth–fronted travelling waves

Here we provide numerical evidence to test the hypothesis that the shape of smooth–fronted travelling waves are given by Equations (19)–(21). Results in Figure S1(a) show a smooth–fronted travelling wave with $c = 2.00$ for $r_1 = r_2 = 1$. Using our long–time numerical PDE solution we plot $U(z)$, $S(z)$ and $W(z)$ at the leading edge of the travelling wave in Figure S1(b). The profiles in Figure S1(b) are a magnified view of the region contained within the purple rectangle in Figure S1(a). At the scale shown in Figure S1(b) we clearly see $U(z)$, $S(z)$ and $W(z)$ decaying to zero with z, and each numerical profile is superimposed with the expressions given by Equations (19)–(21) which match the numerical results extremely well. To further illustrate this point we show an inset in Figure S1(b) comparing the shape of $U(z)$, $S(z)$ and $W(z)$ from the late–time PDE solutions with our proposed asymptotic expressions (19)–(21) for even larger values of z, and again we see an excellent match. While the comparison in Figure S1 is made for one particular choices of r_1, r_2 and c, we also made similar comparisons for different choice of r_1, r_2 and c, and in each case we found an excellent match between Equations (19)–(21) and the shape of the smooth–fronted travelling waves as $z \to \infty$ (not shown).

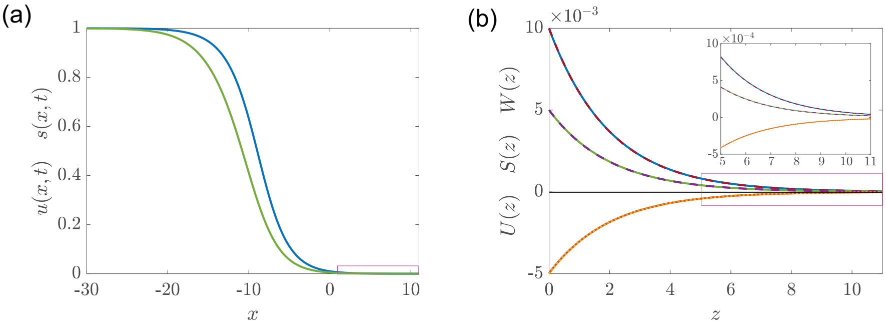

Fig. S1 Shape of the leading edge for smooth–fronted travelling waves as $z \to \infty$. (a) late time numerical solutions of (3)–(4), with initial conditions (8)–(9) and $a = 1/2$. The profile for $u(x,t)$ is shown in blue, and the profile for $s(x,t)$ is shown in green. Parameters in the PDE model are $r_1 = r_2 = 1$ and the long–time speed of the smooth–fronted travelling wave is $c = 2.00$. (b) shows the far-field behaviour of $U(z)$ (blue), $S(z)$ (green) and $W(z)$ (yellow) estimated from the late–time PDE solution superimposed with the solutions given by Equations (19)–(21) in dashed red, dashed purple and dotted red, respectively, for $0 \leq z \leq 11$. The constant C in Equation (19) is obtained by matching the PDE solution with the exponentially decaying solution at $U = 1 \times 10^{-2}$. The solutions in (b) correspond to that part of the solution in (a) contained in the purple rectangle. Similarly, the solution contained in the purple rectangle in (b) is shown as an inset in (b) where the PDE solutions compare very well with the approximate far–field solutions. All numerical PDE solutions correspond to $\Delta x = 1 \times 10^{-2}$, $\Delta t = 1 \times 10^{-3}$ and $\epsilon = 1 \times 10^{-10}$.

S2.2 Phase space and slow manifold

Results in Figure 7 show the phase space and slow manifold for $R = 1$. Analogous results for $R = 0.5$ and $R = 2$ are given here in Figures (S1) and (S2) where we see that the same trends persist for different values of R.

S2.3 Phase space

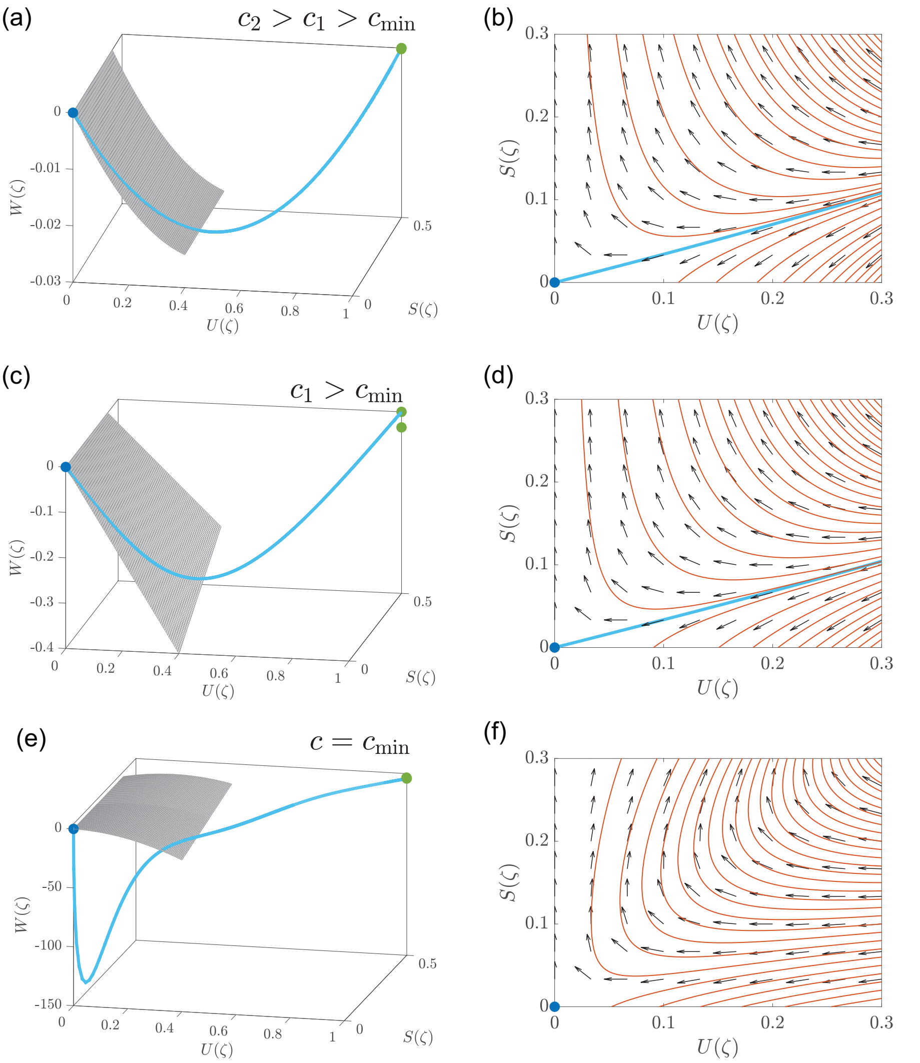

Fig. S2 Desingularised phase space and slow manifold reduction. All results correspond to $R = 0.5$ ($r_1 = 1$, $r_2 = 2$). Results in: (a)–(b) correspond to a smooth–fronted travelling wave with $c_2 = 10$; (c)–(d) correspond to a smooth–fronted travelling wave with $c_1 = 1$; and, (e)–(f) correspond to a sharp–fronted travelling wave with $c_{\min} = 0.27$. Results in the left–most column show the three–dimensional desingularised phase space with the invaded equilibrium point (green dot), the uninvaded equilibrium point (blue dot) and the slow manifold (grey surface). Results in the right–most column show the vector field on the slow manifold, superimposed with several solution trajectories, including the heteroclinic orbit (blue) and several unphysical trajectories (red). The heteroclinic orbit is obtained by solving Equations (3)–(4) numerically with appropriate initial conditions. For (a)–(b) and (c)–(d) the initial conditions are given by Equations (8)–(9) with $a = 1/10$ and $a = 1$, respectively. For (e)–(f) the initial conditions are given by Equations (6)–(7). All numerical PDE solutions correspond to $\Delta x = 1 \times 10^{-4}$, $\Delta t = 1 \times 10^{-3}$ and $\epsilon = 1 \times 10^{-4}$.

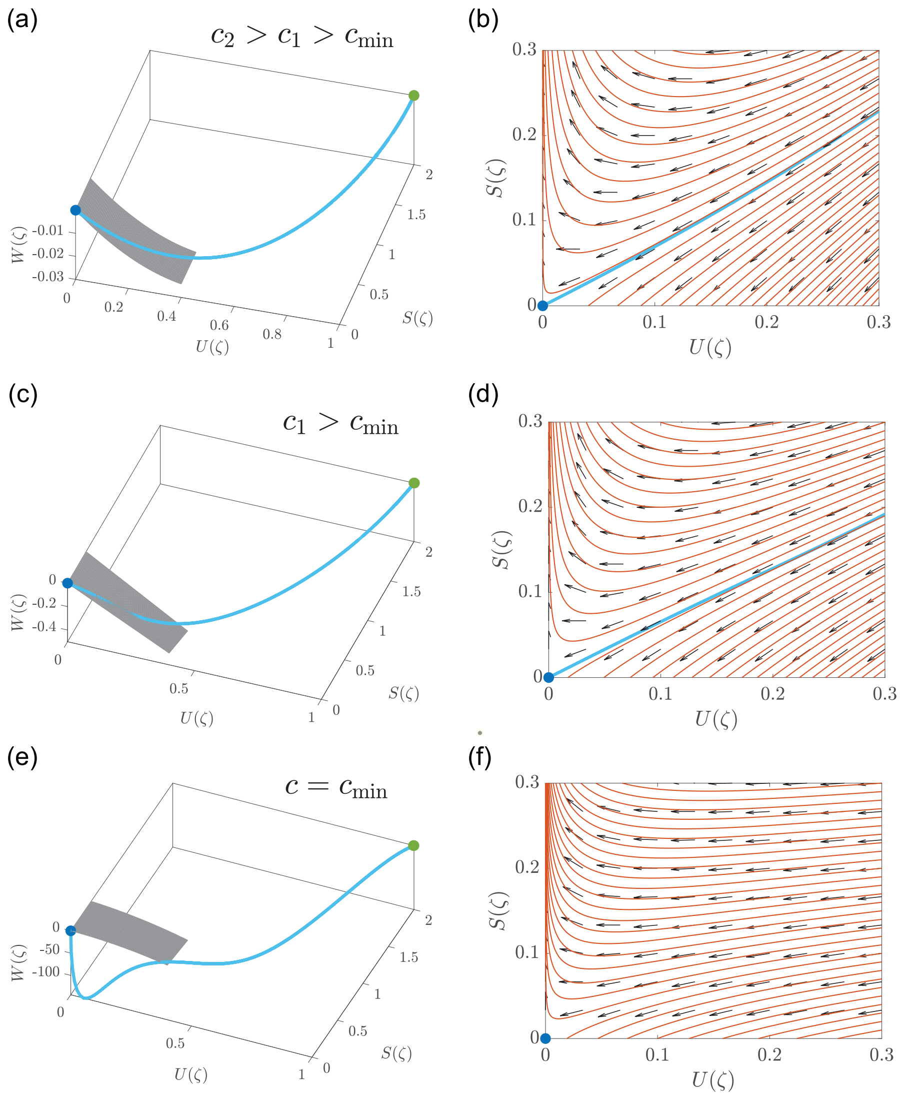

Fig. S3 Desingularised phase space and slow manifold reduction. All results correspond to $R = 2$ ($r_1 = 1$, $r_2 = 0.5$). Results in: (a)–(b) correspond to a smooth–fronted travelling wave with $c_2 = 10$; (c)–(d) correspond to a smooth–fronted travelling wave with $c_1 = 1$; and, (e)–(f) correspond to a sharp–fronted travelling wave with $c_{\min} = 0.30$. Results in the left–most column show the three–dimensional desingularised phase space with the invaded equilibrium point (green dot), the uninvaded equilibrium point (blue dot) and the slow manifold (grey surface). Results in the right–most column show the vector field on the slow manifold, superimposed with several solution trajectories, including the heteroclinic orbit (blue) and several unphysical trajectories (red). The heteroclinic orbit is obtained by solving Equations (3)–(4) numerically with appropriate initial conditions. For (a)–(b) and (c)–(d) the initial conditions are given by Equations (8)–(9) with $a = 1/10$ and $a = 1$, respectively. For (e)–(f) the initial conditions are given by Equations (6)–(7). All numerical PDE solutions correspond to $\Delta x = 1 \times 10^{-4}$, $\Delta t = 1 \times 10^{-3}$ and $\epsilon = 1 \times 10^{-4}$.

S2.4 Phase space for $c < c_{\min}$

In this section we explore the consequences of setting $c < c_{\min}$ in the phase space. Results in Figure S4 show the desingularised phase spaces for $r_1 = r_2 = 1$, where as have previously demonstrated in Figure 7 that late–time numerical solutions of the time–dependent PDE model gives $c_{\min} = 0.29$. Results in Figure S4(a) shows the three–dimensional phase space with $c = 0.40 > c_{\min}$. The yellow trajectory is obtained by integrating (25)–(27) and carefully choosing an initial point to give a heteroclinic orbit that joins the invaded and uninvaded equilibrium points. Figure S4(b) shows a two–dimensional projection of this trajectory in the US plane. Results in Figure S4(c)–(d) show analogous results for $c = 0.29 = c_{\min}$. The most interesting results here are in Figure S4 for $c = 0.25 < c_{\min}$ where we see that the trajectory enters the uninvaded equilibrium point at the origin, but that this trajectory is nonphysical since it involves $U < 0$ along that trajectory. This transition is clear in Figure S4(f) where we show the projection of the trajectory in the US plane. This transition from having $U > 0$ for $c > c_{\min}$ to $U < 0$ for $c < c_{\min}$ also holds for other choices of r_1 and r_2 (not shown).

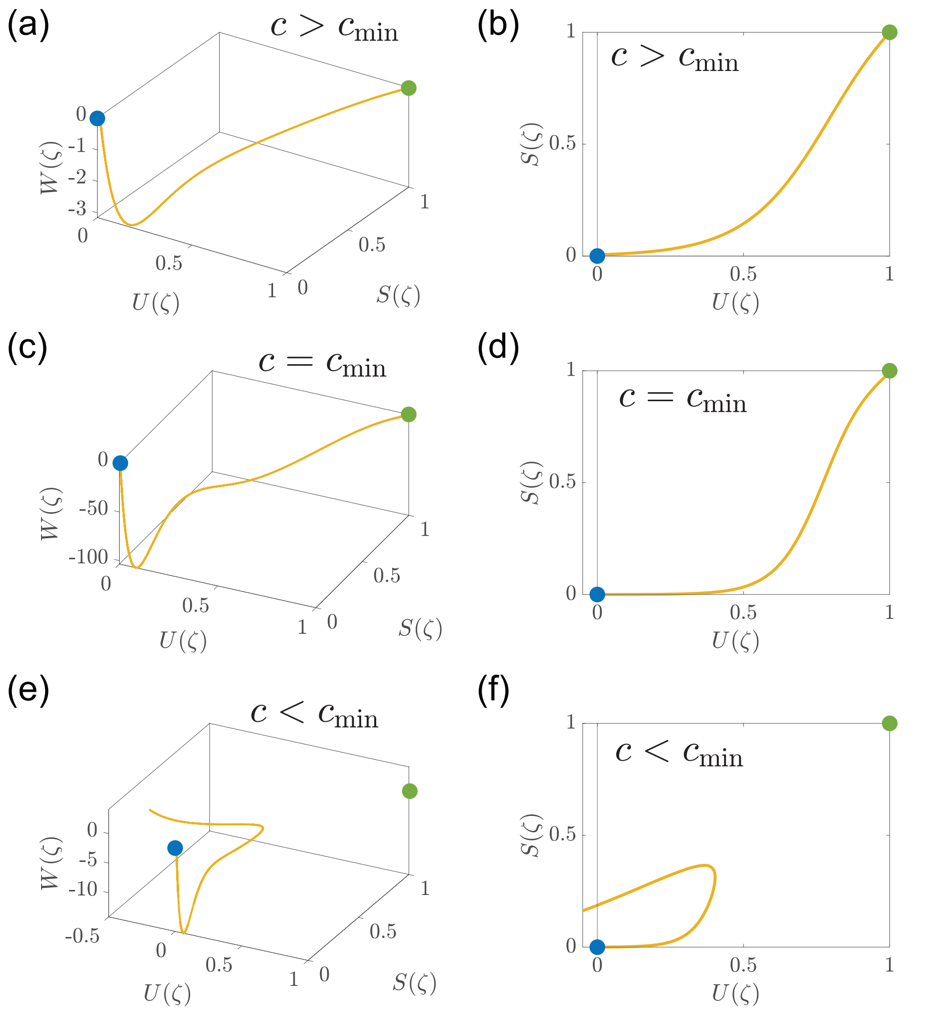

Fig. S4 Phase space for $c < c_{\mathbf{min}}$. All results correspond to $r_1 = 1$ and $r_2 = 2$. Results in: (a)–(b) correspond to a smooth–fronted travelling wave with $c = 0.4 > c_{\text{min}}$; (c)–(d) correspond to a sharp–fronted travelling wave with $c = 0.29 = c_{\text{min}}$; and, (e)–(f) does not correspond to a travelling wave since $c = 0.25 < c_{\text{min}}$. Results in (a), (c) and (e) are obtained by integrating (25)–(27) numerically, with a careful choice of initial condition so that we find the unique trajectory that enters the origin. Results in (b), (d) and (f) are obtained by projecting the three–dimensional trajectory in (a), (c) and (e), respectively, onto the US plane. In each panel the invaded equilibrium point, $(\bar{U}, \bar{S}, \bar{W}) = (1, R, 0)$, is shown with a green disc. The uninvaded equilibrium point, $(\bar{U}, \bar{S}, \bar{W}) = (0, 0, 0)$, is shown with a blue disc. All trajectories are obtained by integrating (25)–(27) using Heun's method with $\mathrm{d}\zeta = 1 \times 10^{-2}$.

S2.5 Perturbation solution for $c \to \infty$

In Equation (52) we left the expression for $U_1(\hat{z})$ as an integral. Here, we give present a solution for $U_1(\hat{z})$ for some special choices. For $r_2 = 1$ we obtain

$$U_1(\hat{z}) = \left(\frac{r_1 \exp(\hat{z})}{[1+\exp(\hat{z})]^2}\right)\Big(-\ln[1+\exp(\hat{z})]^2 + 2\hat{z}\ln[1+\exp(\hat{z})] + 2\mathrm{Li}_2[1+\exp(\hat{z})] - \ln[\exp(-\hat{z})+1]\Big)\,, \tag{S9}$$

and when $r_2 = 2$ we obtain

$$U_1(\hat{z}) = \left(\frac{r_1 \exp(\hat{z})}{2[1+\exp(\hat{z})]^2}\right)\Big(-2\ln[1+\exp(\hat{z})]^2 + 4\hat{z}\ln[1+\exp(\hat{z})] + 4\mathrm{Li}_2[1+\exp(\hat{z})] + [\exp(2\hat{z}) + 4\exp(\hat{z}) - 1]\ln[\exp(-\hat{z})+1] - \exp(\hat{z})\Big)\,, \tag{S10}$$

where $\mathrm{Li}_2(x)$ is a special function called the dilogarithm function that is given by (Maple 2021)

$$\mathrm{Li}_2(x) = \int_1^x \frac{\ln(t)}{1-t}\,\mathrm{d}t. \tag{S11}$$

Additional results in Figure (S5) compare the $\mathcal{O}(1/c^2)$ perturbation solution for $U(z)$ with numerical estimates from the long–time numerical PDE solution, together with the $\mathcal{O}(1)$ perturbation solution for $S(z)$ with numerical estimates from the long–time numerical PDE solution. In this case we focus on $c = 1$ for various values of r_1 and r_2, as indicated. Here, despite the fact we are working with a relatively small value of c and the perturbation solutions are valid in the limit $c \to \infty$, the accuracy of the perturbation solutions is remarkable.

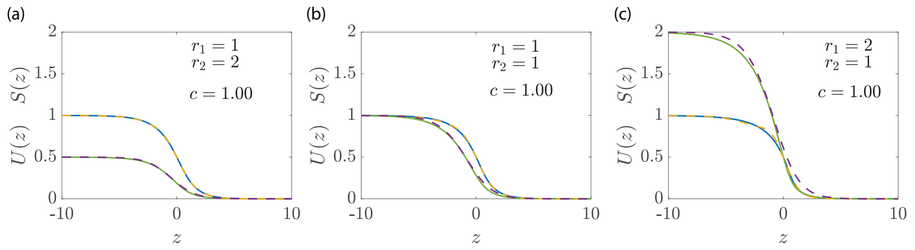

Fig. S5 Perturbation solution for smooth–fronted travelling wave solutions. Travelling wave solutions $U(z)$ and $S(z)$ are obtained from solving Equations (6)–(8) with initial conditions (11)–(12), where $a = 1$ such as the obtained wave speed is $c = 1.00$, with $R = 0.5, 1$ and 2 in (a)–(c), respectively. Numerical solutions $U(z)$ and $S(z)$ are shown in blue and green, respectively, and perturbation solutions for $U(z)$ and $S(z)$ are shown in dashed yellow and purple, respectively.

References

The MathWorks Inc. (2021) Quiver. Retrieved November 2021 from https://www.mathworks.com/help/matlab/ref/quiver.html.

Maplesoft, a division of Waterloo Maple Inc. (2021) Maple User Manual: Dilog. Retrieved November 2021 from https://www.maplesoft.com/support/help/Maple/view.aspx?path=dilog.
